# Parental migration, socioeconomic deprivation and hospital admissions in preschool children in England: national birth cohort study, 2008 to 2014

**DOI:** 10.1186/s12916-024-03619-1

**Published:** 2024-09-27

**Authors:** Kate M. Lewis, Rachel Burns, Mario Cortina-Borja, Anja Heilmann, Alison Macfarlane, Selina Nath, Sarah M. Salway, Sonia Saxena, Nazmy Villarroel-Williams, Russell Viner, Pia Hardelid

**Affiliations:** 1 Great Ormond Street Institute of Child Health, University College London, London, UK; 2 Institute of Health Informatics, University College London, London, UK; 3 Department of Epidemiology and Public Health, University College London, London, UK; 4 Centre for Maternal and Child Health Research, City, University of London, London, UK; 5 Department of Sociological Studies, University of Sheffield, Sheffield, UK; 6 School of Public Health, Imperial College London, London, UK; 7 Department of Psychology, Sociology and Politics, Sheffield Hallam University, Sheffield, UK

**Keywords:** Migration, United Kingdom, Health services, Hospital admissions, Child health

## Abstract

**Background:**

A third of children born in England have at least one parent born outside the United Kingdom (UK), yet family migration history is infrequently studied as a social determinant of child health. We describe rates of hospital admissions in children aged up to 5 years by parental migration and socioeconomic group.

**Methods:**

Birth registrations linked to Hospital Episode Statistics were used to derive a cohort of 4,174,596 children born in state-funded hospitals in England between 2008 and 2014, with follow-up until age 5 years. We looked at eight maternal regions of birth, maternal country of birth for the 6 most populous groups and parental migration status for the mother and second parent (UK-born/non-UK-born). We used Index of Multiple Deprivation (IMD) quintiles to indicate socioeconomic deprivation. We fitted negative binomial/Poisson regression models to model associations between parental migration groups and the risk of hospital admissions, including interactions with IMD group.

**Results:**

Overall, children whose parents were both born abroad had lower emergency admission rates than children with parents both born in the UK. Children of UK-born (73.6% of the cohort) mothers had the highest rates of emergency admissions (171.6 per 1000 child-years, 95% confidence interval (CI) 171.4–171.9), followed by South Asia-born mothers (155.9 per 1000, 95% CI 155.1–156.7). The high rates estimated in the South Asia group were driven by children of women born in Pakistan (186.8 per 1000, 95% CI 185.4–188.2). A socioeconomic gradient in emergency admissions was present across all maternal regions of birth groups, but most pronounced among children of UK-born mothers (incidence rate ratio 1.43, 95% CI 1.42–1.44, high vs. low IMD group). Patterns of planned admissions followed a similar socioeconomic gradient and were highest among children with mothers born in Middle East and North Africa, and South Asia.

**Conclusions:**

Overall, we found the highest emergency admission rates among children of UK-born parents from the most deprived backgrounds. However, patterns differed when decomposing maternal place of birth and admission reason, highlighting the importance of a nuanced approach to research on migration and health.

**Supplementary Information:**

The online version contains supplementary material available at 10.1186/s12916-024-03619-1.

## Background

International migration, defined here as the movement of people to countries outside their place of birth, is a growing feature of our modern globalised world [1]. A third (34.2%) of children born in England and Wales in 2021 had at least one parent born outside the United Kingdom (UK) [2]. People who migrate can face challenges navigating new health and social care services for themselves and their children, which, for some migrant groups in particular, may operate within legislation hostile to their presence [3]. In conjunction with other interlinking social determinants of health, parental migration is, therefore, an important topic of public health inquiry [1, 4–6]. However, despite the size of the population of children born to parents who are international migrants, and the increased propensity for children with parents born outside the UK to be living in poverty, [7] there is no national-level research on the health and healthcare utilisation of children born to parents who have migrated in the UK [8]. This reflects the predominant focus on ethnicity, rather than migration history or country of birth, as a determinant of health in the UK and, relatedly, the lack of recording of migration status in routine health data [6].

A systematic review on health service use, mostly conducted in North America and Europe (but including no UK-based studies), identified higher hospital and emergency service use among first- and second-generation migrant children compared with the rest of the childhood population [5]. This suggests that migrants and their children face barriers in accessing appropriate and timely healthcare. However, differences in the structural and political context of destination countries, the health system and composition of the migrant population, limit the generalisability of these findings to the UK. Furthermore, as highlighted in UK-based research on maternal and perinatal outcomes, [9–11] the experience of these systems by international migrants and their children is not homogenous. Whilst primary and emergency care is free of charge for everyone in the UK, [12] barriers to accessing health services, such as unfamiliarity with the UK system, a lack of language support, discrimination and childcare and transport costs[13, 14], may be differentially weighted across groups. It is therefore important to conduct analyses at a level beyond a dichotomous measure of migration.

Drawing on novel linkage of national birth registration and hospital admission data, the aim of this study was to describe the association between parental migration (defined as maternal world region/country of birth and parental migration status) and rates of early childhood hospital admissions in England, and how this association varies by socioeconomic circumstances. Our particular focus was on maternal place of birth. Elucidating patterns of secondary healthcare use for young children with different family histories of migration, particularly at the intersection with socioeconomic deprivation—a documented risk factor for emergency hospital use in the general population in the UK, [15, 16] is a key step towards tailored and appropriate action on unmet need and health inequities. We compare rates of emergency and planned hospital admissions overall and for three common childhood conditions (acute infections, feeding difficulties and jaundice and tooth extractions for caries), which are considered preventable with the appropriate care provision.

## Methods

### Data sources and linkage

To conduct this population-based cohort study, we used linked de-identified Office for National Statistics (ONS) birth and death registration data, National Health Service (NHS) birth notification data and NHS Hospital Episode Statistics Admitted Patient Care (hereafter referred to as “HES”) records from England (Additional File 1: Table S1). ONS birth and death registrations (for children who die up to the age of 15 years), and ONS birth registration and NHS notifications, are routinely linked by ONS using deterministic matching algorithms [17, 18]. HES contains information on NHS-funded inpatient stays in hospitals in England, and captures about 97% of all births (and associated deliveries) in England, with additional details about the birth/delivery appended to the core record in a “maternity tail” [19]. In this study, we used HES infant birth records linked to longitudinal hospital admissions via the child’s pseudonymised patient identifier (“HESID”). We also used HES maternal delivery records to provide additional background characteristics.

Linkage between birth registrations-notifications and HES was carried out by NHS Digital in partnership with the ONS and City, University of London as part of a previous NIHR funded study [18, 20]. Briefly, the datasets were linked by HSCIC (now NHS England) using an adapted version of the inhouse deterministic stepwise linkage algorithm routinely used to link HES and ONS death registrations. For this study, we additionally had national data opt-outs applied to HES records [21]. Birth registration-HES infant birth records were linked using NHS number, date of birth, postcode and sex, whilst birth registration-HES maternal delivery records were linked using a larger number of identifiers but without NHS numbers [20]. In this study, 91.9% of birth registrations had a linked HES infant birth record (see “ Results” section) and 86.1% had a linked HES maternal delivery record.

### Study cohort

We included live births between 1st January 2008 and 31st December 2014 in England. Infants were excluded if they were as follows: from a multiple birth (identified using birth registration records) because of an increased risk of false matches in HES records; [22] born to mothers not resident in England (identified using resident postcode at delivery on birth registration records) to prevent systematic loss to follow-up; or born in a military or private maternity unit (identified using the maternity unit identifier on birth registration records) as these children did not have accompanying HES birth record. Birth registrations unlinked to a HES birth record were also excluded in our study, as the cohort had been configured such that babies without a HES birth record did not have a link to HES admission data; therefore, these children cannot be followed up. Home births were included in this study if they had linked birth registration-HES birth records. Finally, children with missing place of residence, missing maternal place of birth, or with ≤ 1 day follow-up were excluded.

### Outcomes and follow-up

There were five hospital admission-based outcomes in this study (Table 1). We defined an admission as one continuous stay at a hospital, including admissions within 1 day of each other and transfers between hospitals [22]. We first examined incidence rates of admissions up to age 5 years irrespective of diagnosis, stratified by admission method (emergency or planned). Emergency admissions are defined by NHS England as “unpredictable and at short notice because of clinical need” and planned (or elective) admissions defined as occurring where “the decision to admit could be separated in time from the actual admission” [23]. Our secondary outcomes were three common causes of paediatric hospital admissions that may be avoidable with preventative or responsive primary/community care: [24–26] emergency admissions for acute infections, emergency admissions for feeding problems or jaundice; and planned admissions for tooth extraction due to caries. Reducing childhood admissions for lower respiratory tract infections and tooth extractions for caries have been specifically identified by the Department of Health and Social Care as priorities within the NHS Outcomes Framework [2]. Hospital admissions due to neonatal feeding problems may be preventable with feeding advice and support, in the immediate post-neonatal period, either in the hospital after delivery, or at home [3].

**Table 1:** Case definitions and follow-up time for each study outcome

Outcome	Primary diagnosis		Admission method^a^	Follow-up	
	**Conditions**	**ICD-10 codes**		**Start**	**End**^b^
All planned admissions	Any	Any	Planned	Discharge from birth admission	5^th^ birthday
All emergency admissions	Any	Any	Emergency	Discharge from birth admission	5^th^ birthday
Acute infections	Lower respiratory tract infection, upper respiratory tract infection, urinary tract infection, dehydration, or gastroenteritis	See Additional File 1: Table S2	Emergency	Discharge from birth admission	5^th^ birthday
Neonatal feeding difficulties and jaundice	Neonatal jaundice from other and unspecified causes, or feeding problems of new-born	P59, P92	Emergency	Discharge from birth admission	6 months of age
Tooth extractions due to caries	Dental caries AND a main operative procedure of surgical removal of tooth, or simple extraction of tooth	K02, K04, OPCS-4 codes F09, F10	Planned	2^nd^ birthday	5^th^ birthday

*ICD-10* International Classification of Diseases 10th Revision, *OPCS-4 * Office of Population Censuses and Surveys codes for interventional procedures

^a^ determined by admission method for the first episode in admission

^b^ end to follow-up was at the age specified in the table, date of death, the end of the study (31 December 2014) or estimated emigration date (set as the mid-point between the child’s date of birth and the date at which a hospital admission with a non-English address occurred during follow-up), whichever came first

Follow-up began on the day after discharge from birth admission or age 2 years (for the tooth extraction outcome). Follow-up ended on the earliest of 5 years of age (or 6 months for feeding problems or jaundice), date of death, the end of the study (31 December 2014) or estimated emigration date (set as the mid-point between the child’s date of birth and the date at which a hospital admission with a non-English address occurred during follow-up). The estimated emigration date defined here is only available for children with a hospital record in an NHS-funded hospital in England after emigration and, therefore, only captures a minority of emigration from England. We ran additional analyses to check the robustness of results different scenarios accounting for a wider definition of emigration (see sensitivity analyses). The maximum follow-up date available in this linked dataset was 31st December 2014.

### Exposures

**Fig. 1:**
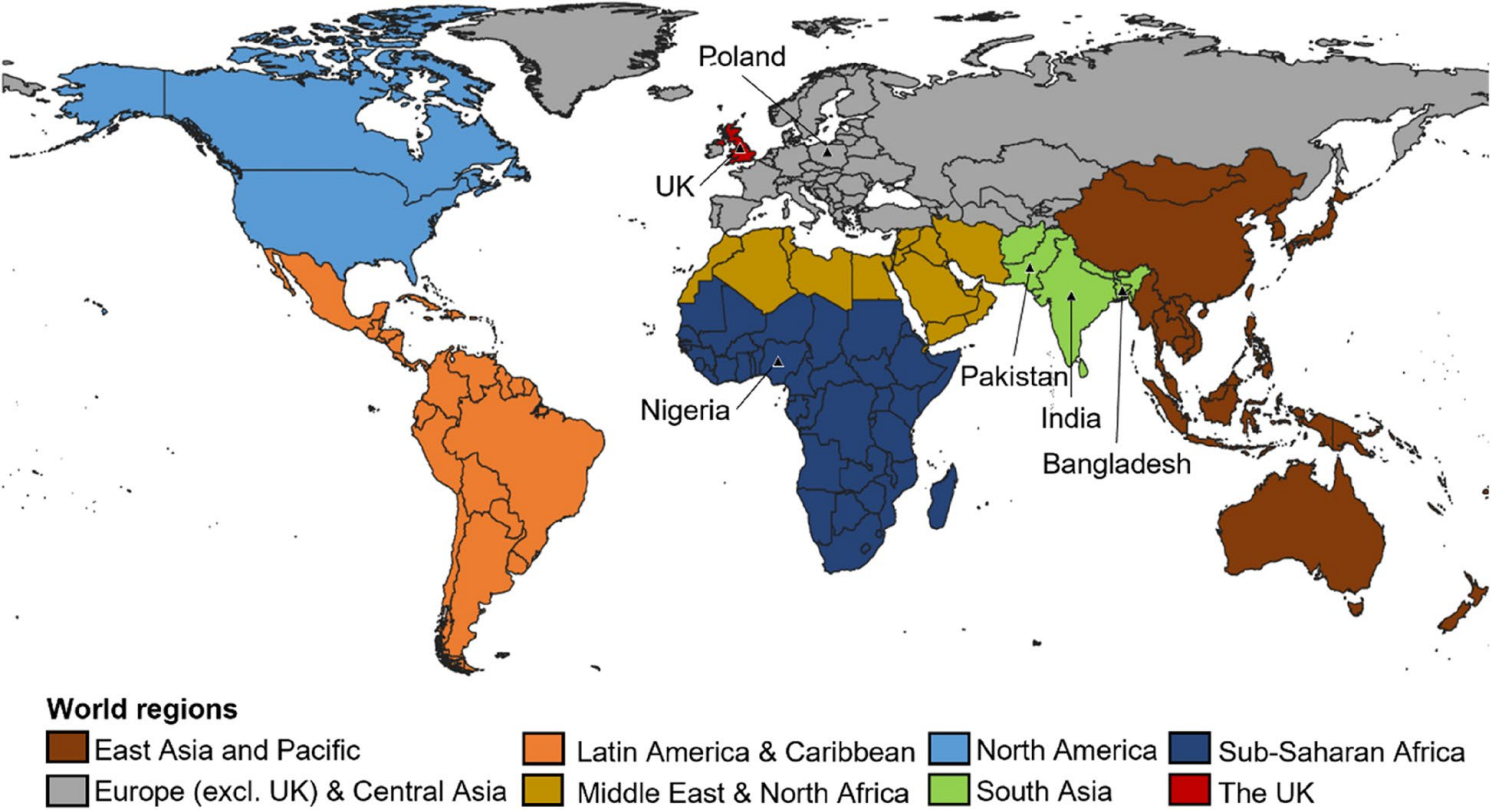
Categories of maternal world regions of birth (with countries of birth indicated by the triangular symbols)

The primary exposure in this study was maternal world region of birth, derived from mother’s country of birth recorded at birth registration. Countries were harmonised using the ONS’s National Statistics country classification, as specified in 2015, which is based on ISO 3166–1 (the international standard for country codes) [27]. Country of birth was then categorised into eight groups, adapted from the World Bank’s 7-group classification of geographical regions (separating the UK from the Europe and Central Asia group, see Fig. 1) [28]. Whilst we acknowledge that parents moving to the UK from different countries within the same global region may have very different health profiles and experiences of healthcare in England, we use World Bank regional groups to enable broad descriptions of the whole population, whilst improving on previous studies which have used dichotomous groupings.

We also present results by maternal countries of birth for a subset of the sample; the six most common countries in our dataset (Bangladesh, India, Nigeria, Pakistan, Poland and the UK). We conceptualise country of birth as a broad indicator of pre-migration and transition circumstances, as well as cultural identity (including language and religion) [1]. For comparison with previous work, a six-category parental “migration status” variable was also created as a secondary exposure. This was defined by mothers’ and second parents’ countries of birth grouped into UK, non-UK and sole mother registration, where second parent information was not recorded.

### Covariates

Socioeconomic deprivation was measured using the Index of Multiple Deprivation (IMD) 2010 [29]. IMD is constructed using seven domains of deprivation (income, employment, education and skills, health and disability, crime, barriers to housing and services, and living environment) calculated at the Lower layer Super Output Area (LSOA) level, an area with an average population of 1500 residents or 650 households. Scores are weighted and ranked to produce a relative measure of deprivation for each small area across England. In this study, IMD is based on the LSOA of each child’s residential postcode at delivery from birth registration data (supplemented by HES birth or delivery records if missing), and is split into 5 groups (from most to least deprived). An updated IMD measure is available every 3 to 5 years; we used IMD 2010 to use a consistent measure throughout analyses. Whilst IMD is a small-area level measure of deprivation, we used it as an indicator of family-level socioeconomic position in this study (a common approach to health disparities research in the UK) [30].

We included year of birth, defined using each child’s date of admission in their HES birth record. We also selected the following variables to be summarised given their association with migration and/or hospital admission rates as shown in the research literature: HES coded maternal ethnicity, child’s sex recorded by physician at birth, region of residence, hospital record-identified congenital anomaly and maternal age (for definitions see Additional File 1: Tables S3-S4) [1, 26, 31, 32].

### Statistical methods

We summarised the distribution of key childhood characteristics by maternal world region of birth. Observed incidence rates of hospital admissions for children within the cohort were then calculated by dividing the number of admissions by person-time at risk per 1000 child-years.

To examine whether incidence rates of admissions varied by maternal world region of birth and IMD group, we fitted separate regression models for each outcome with the logarithm of person-time as the offset. We used negative binomial or Poisson regression models depending on the presence of overdispersion in each model as indicated by Pearson’s dispersion statistic. To account for unmeasured shared factors of children in the cohort with the same mother, we used robust standard errors clustered at the family level. Alongside maternal world region of birth and IMD group, we adjusted for year of birth to account for likely cohort effects in the data including changing population composition, thresholds for hospital admission and infection risk [33]. We did not include other available covariates in the model (such as child sex and maternal age at birth) as we hypothesise that they occur temporally after the exposure and therefore cannot be confounders of the relationship between parental migration and hospital admission (see Additional File 2: Figure S1).

To examine the interaction between maternal world region of birth and IMD groups we ran models with and without an interaction term, comparing models’ goodness-of-fit using Akaike’s information criterion value (AIC; with a smaller value indicating preferable model fit). We estimated the adjusted incidence rate ratios (IRRs) of admission for each IMD group in comparison to the least deprived IMD group, within maternal region groups. We further estimated marginal incidence rates of hospital admissions for each level of the interaction between maternal region of birth and IMD group, with year of birth set to mid-study (2011). We repeated the above analysis, including interactions with IMD, by maternal country of birth and parental migration status.

Two sensitivity analyses were performed. We repeated the above analyses stratifying the cohort by London or non-London region of birth to account for known geographical variability in migrant populations and incidence rates of hospital admissions, particularly in differential thresholds for admissions [31]. Secondly, since data on embarkations from the UK were not available and very few emigrations are captured in HES, we ran simulations to assess how different levels of international emigration could affect the final results (see Additional File 3).

Statistical analyses were conducted in Stata v16 within the ONS Secure Research Service.

### Patient and public involvement

A group of women (*n* = 7), born in a range of countries outside the UK, who had children whilst living in the UK, took part in a group discussion about our research proposal at the beginning stages of this project. The group supported the project aims, particularly the focus on maternal place of birth rather than a binary UK/non-UK born split, and offered insight into potential mechanisms for different outcomes, such as culturally relevant services. Participants were identified by word of mouth and compensated for their time by a gift voucher. We did not require ethics review to conduct this involvement activity as parents were advisors to the study team rather than research subjects.

### Role of the funding source

The funders of the study had no role in study design, data collection, data analysis, data interpretation, or writing of the report. Due to restrictions imposed by the data providers, only KML, SN and PH had access to the raw data. All authors accept responsibility to submit for publication.

## Results

**Fig. 2:**
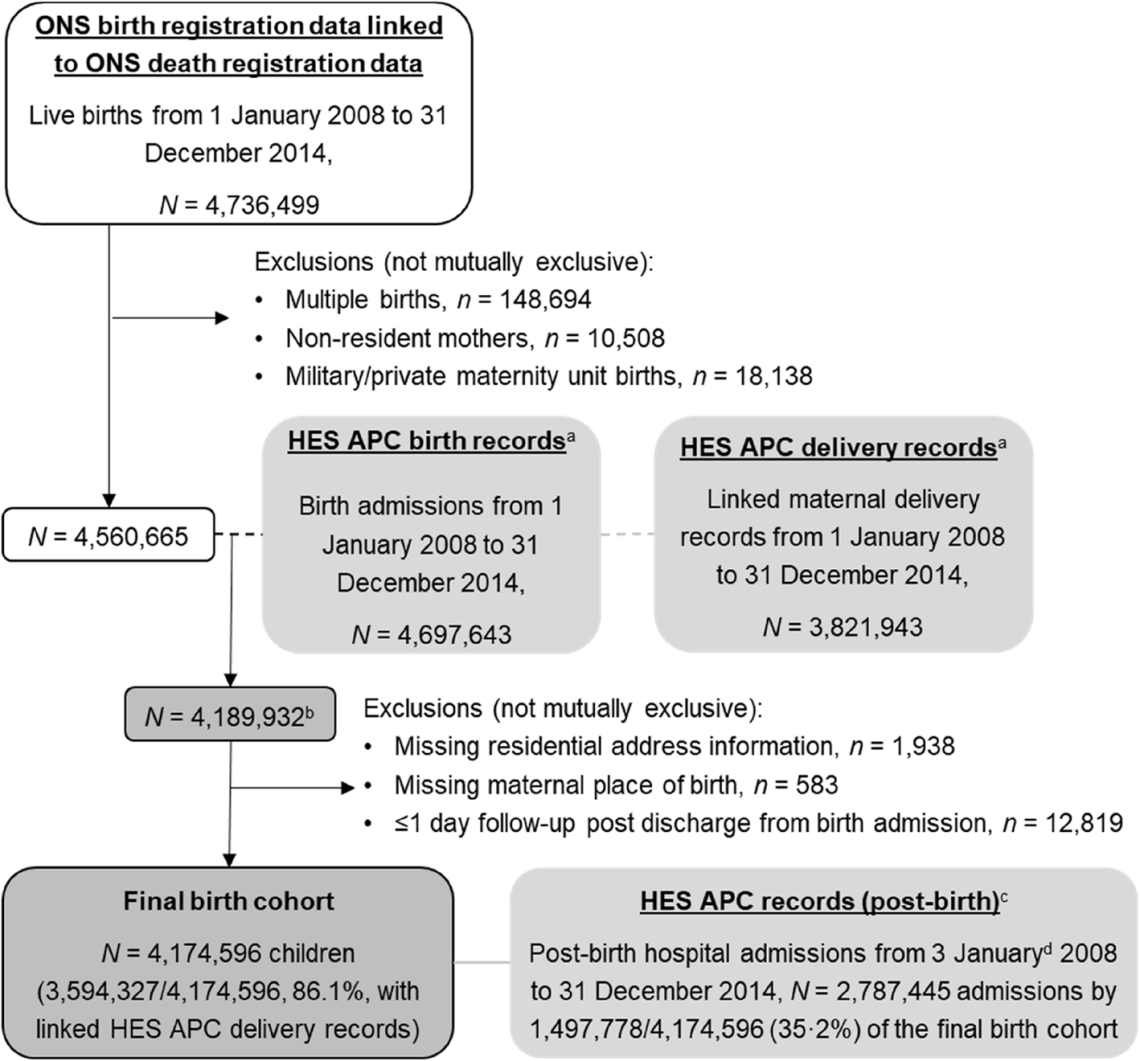
Flow chart showing study sample derivation. HES APC = Hospital Episode Statistics Admitted Patient Care, ONS = Office for National Statistics; ^a^Data opt outs applied (digital.nhs.uk/services/national-data-opt-out); ^b^Linkage with 91·9% of ONS birth registrations after exclusions; ^c^Any admission to hospital after discharge from birth admission to 5 years of age within study period; ^d^As follow-up starts a day after discharge from birth admission, the earliest post-birth hospital admission is 3^rd^ January 2008

There were 4,736,499 live births in England between 1 January 2008 and 31 December 2014 recorded in the ONS birth registration dataset. As shown in Fig. 2, 175,834 (3.7%) of these births met at least one of the predefined exclusion criteria and an additional 370,730 (8.1%) birth registrations were also excluded as they did not have a linked HES birth record. Patterns of the unlinked 8.1% HES birth records differed across child characteristics and were particularly common in earlier study years (14.9% in 2008 vs 6.2% in 2014); children with mothers born in North America (9.6%), Latin America and Caribbean (9.2%) and Sub-Saharan Africa (9.1%); children without joint birth registrations (12.6% for UK-born mothers and 9.9% for non-UK-born-mothers); and the North West of England (9.7% compared to 3.8% in North East; see Additional File 4: Table S7). Lastly, 15,336 (0.4%) linked records were excluded due to missing information on residential address, mothers place of birth or $\le$1 day of follow-up time.

### Cohort characteristics

The final cohort included 4,174,596 children (48.7% female), of whom 1,100,827 (26.4%) had mothers born outside the UK. The most common maternal countries of birth, after the UK, were Poland (122,889; 2.9% of the cohort), Pakistan (115,886; 2.8%), India (85,830; 2.1%), Bangladesh (50,831; 1.2%) and Nigeria (42,934, 1.0%; Additional File 5: Table S8). A non-UK-born second parent was recorded for 1,025,992 (24.6%) children and 233,864 (5.6%) had no second parent recorded on their birth registration (6.1% with UK-born mothers compared to 4.1% with non-UK-born mothers, Table 2). In total, 1,292,853 children (31.0% of those with records for two parents) had at least one parent born outside the UK. More than 40% of children with mothers born in Sub-Saharan Africa, the Middle East and North Africa, and South Asia were in the most deprived IMD group, compared to 24.5% whose mothers were born in the UK. 11.0% of children with mothers born in the UK had a London-based residence at birth, compared with 36.3–59.9% of mother born outside the UK. Mothers born in East Asia and Pacific, and North America tended to be older at delivery, and the proportion of children with a hospital record-identified congenital anomaly was highest in those with mothers born in South Asia, followed by the UK (2.8% and 2.7%, respectively).

**Table 2:** Key characteristics of final study cohort, by maternal world region of birth

	East-Asia & Pacific	Europe & Central Asia	Latin America & Caribbean	Middle East & North Africa	North America	South Asia	Sub-Saharan Africa	UK	Total
Total *N*	94,158	380,504	40,413	60,820	22,534	296,976	205,422	3,073,769	4,174,596
Average follow up (years)	3.22	2.99	3.19	3.12	3.15	3.15	3.24	3.19	3.17
	***N* (%)**	***N* (%)**	***N* (%)**	***N* (%)**	***N* (%)**	***N* (%)**	***N* (%)**	***N* (%)**	***N* (%)**
SP place of birth									
UK	37,227 (39.5)	98,791 (26.0)	12,351 (30.6)	7656 (12.6)	14,511 (64.4)	64,931 (21.9)	31,394 (15.3)	2,647,879 (86.1)	2,914,740 (69.8)
Non-UK	54,746 (58.1)	264,876 (69.6)	24,624 (60.9)	52,110 (85.7)	7663 (34.0)	229,779 (77.4)	154,718 (75.3)	237,476 (7.7)	1,025,992 (24.6)
No SP registered	2185 (2.3)	16,837 (4.4)	3438 (8.5)	1054 (1.7)	360 (1.6)	2266 (0.8)	19,310 (9.4)	188,414 (6.1)	233,864 (5.6)
Year of birth									
2008	12,992 (13.8)	42,017 (11.0)	5532 (13.7)	7527 (12.4)	2942 (13.1)	39,587 (13.3)	29,338 (14.3)	410,573 (13.4)	550,508 (13.2)
2009	13,462 (14.3)	47,054 (12.4)	5903 (14.6)	8545 (14.0)	3081 (13.7)	41,158 (13.9)	30,113 (14.7)	434,200 (14.1)	583,516 (14.0)
2010	13,987 (14.9)	52,493 (13.8)	5859 (14.5)	8835 (14.5)	3283 (14.6)	41,600 (14.0)	30,573 (14.9)	452,316 (14.7)	608,946 (14.6)
2011	13,631 (14.5)	54,812 (14.4)	5711 (14.1)	8495 (14.0)	3254 (14.4)	43,580 (14.7)	29,737 (14.5)	455,068 (14.8)	614,288 (14.7)
2012	14,491 (15.4)	59,260 (15.6)	5854 (14.5)	8986 (14.8)	3353 (14.9)	45,172 (15.2)	30,043 (14.6)	457,820 (14.9)	624,979 (15.0)
2013	12,825 (13.6)	61,065 (16.0)	5751 (14.2)	9149 (15.0)	3262 (14.5)	43,879 (14.8)	28,510 (13.9)	436,532 (14.2)	600,973 (14.4)
2014	12,770 (13.6)	63,803 (16.8)	5803 (14.4)	9283 (15.3)	3359 (14.9)	42,000 (14.1)	27,108 (13.2)	427,260 (13.9)	591,386 (14.2)
IMD group									
1 Least deprived	15,269 (16.2)	42,101 (11.1)	3859 (9.5)	4142 (6.8)	5288 (23.5)	15,405 (5.2)	15,866 (7.7)	527,475 (17.2)	629,405 (15.1)
2	15,904 (16.9)	52,849 (13.9)	4393 (10.9)	5922 (9.7)	5385 (23.9)	22,930 (7.7)	17,213 (8.4)	551,502 (17.9)	676,098 (16.2)
3	18,775 (19.9)	73,994 (19.4)	6532 (16.2)	9085 (14.9)	4933 (21.9)	45,595 (15.4)	26,627 (13.0)	591,164 (19.2)	776,705 (18.6)
4	22,558 (24.0)	102,260 (26.9)	11,466 (28.4)	15,083 (24.8)	4395 (19.5)	84,321 (28.4)	54,357 (26.5)	649,457 (21.1)	943,897 (22.6)
5 Most deprived	21,652 (23.0)	109,300 (28.7)	14,163 (35.0)	26,588 (43.7)	2533 (11.2)	128,725 (43.3)	91,359 (44.5)	754,171 (24.5)	1,148,491 (27.5)
Region of residence									
North east	2536 (2.7)	6256 (1.6)	259 (0.6)	1996 (3.3)	489 (2.2)	5237 (1.8)	2558 (1.2)	174,776 (5.7)	194,107 (4.6)
North west	7935 (8.4)	27,196 (7.1)	1344 (3.3)	6846 (11.3)	1368 (6.1)	33,309 (11.2)	13,710 (6.7)	444,311 (14.5)	536,019 (12.8)
Yorkshire & humber	5516 (5.9)	25,459 (6.7)	984 (2.4)	6334 (10.4)	1241 (5.5)	29,842 (10.0)	9683 (4.7)	338,056 (11.0)	417,115 (10.0)
East midlands	4386 (4.7)	28,071 (7.4)	1195 (3.0)	3344 (5.5)	1080 (4.8)	16,275 (5.5)	10,469 (5.1)	277,173 (9.0)	341,993 (8.2)
West midlands	6245 (6.6)	26,517 (7.0)	2921 (7.2)	6680 (11.0)	1002 (4.4)	42,195 (14.2)	14,998 (7.3)	339,334 (11.0)	439,892 (10.5)
East of england	8992 (9.5)	43,473 (11.4)	2710 (6.7)	3123 (5.1)	2497 (11.1)	20,429 (6.9)	15,115 (7.4)	346,368 (11.3)	442,707 (10.6)
London	35,788 (38.0)	138,083 (36.3)	24,223 (59.9)	25,025 (41.1)	8836 (39.2)	111,196 (37.4)	107,417 (52.3)	337,857 (11.0)	788,425 (18.9)
South east	16,023 (17.0)	58,048 (15.3)	4640 (11.5)	5338 (8.8)	4301 (19.1)	31,634 (10.7)	23,331 (11.4)	499,489 (16.3)	642,804 (15.4)
South west	6737 (7.2)	27,401 (7.2)	2137 (5.3)	2134 (3.5)	1720 (7.6)	6859 (2.3)	8141 (4.0)	316,405 (10.3)	371,534 (8.9)
Maternal ethnic group									
Bangladeshi	58 (0.1)	69 (< 0.1)	8 (< 0.1)	113 (0.2)	10 (0.0)	36,575 (12.3)	109 (0.1)	10,023 (0.3)	46,965 (1.1)
Indian	616 (0.7)	425 (0.1)	135 (0.3)	740 (1.2)	281 (1.2)	64,014 (21.6)	4045 (2.0)	36,985 (1.2)	107,241 (2.6)
Pakistani	244 (0.3)	1001 (0.3)	14 (< 0.1)	1446 (2.4)	113 (0.5)	86,836 (29.2)	609 (0.3)	49,968 (1.6)	140,231 (3.4)
Black african	132 (0.1)	1364 (0.4)	543 (1.3)	3555 (5.8)	110 (0.5)	376 (0.1)	89,801 (43.7)	8026 (0.3)	103,907 (2.5)
Black caribbean	44 (< 0.1)	193 (0.1)	9629 (23.8)	50 (0.1)	111 (0.5)	48 (< 0.1)	1669 (0.8)	14,863 (0.5)	26,607 (0.6)
White british	9040 (9.6)	35,577 (9.3)	1866 (4.6)	2142 (3.5)	5336 (23.7)	2040 (0.7)	11,747 (5.7)	2,232,271 (72.6)	2,300,019 (55.1)
White other	9461 (10.0)	212,598 (55.9)	7393 (18.3)	6443 (10.6)	8761 (38.9)	1643 (0.6)	10,783 (5.2)	56,579 (1.8)	313,661 (7.5)
Other	50,530 (53.7)	33,285 (8.7)	9375 (23.2)	30,646 (50.4)	1803 (8.0)	43,852 (14.8)	33,258 (16.2)	67,878 (2.2)	270,627 (6.5)
Not known or missing	24,033 (25.5)	95,992 (25.2)	11,450 (28.3)	15,685 (25.8)	6009 (26.7)	61,592 (20.7)	53,401 (26.0)	597,176 (19.4)	865,338 (20.7)
Maternal age									
< 20	866 (0.9)	9797 (2.6)	1195 (3.0)	769 (1.3)	210 (0.9)	2153 (0.7)	3220 (1.6)	192,498 (6.3)	210,708 (5.0)
20–24	6509 (6.9)	56,946 (15.0)	4483 (11.1)	8899 (14.6)	1300 (5.8)	48,360 (16.3)	22,830 (11.1)	615,649 (20.0)	764,976 (18.3)
25–29	19,486 (20.7)	121,150 (31.8)	8915 (22.1)	19,568 (32.2)	4399 (19.5)	108,930 (36.7)	56,274 (27.4)	829,993 (27.0)	1,168,715 (28.0)
30–34	34,378 (36.5)	121,917 (32.0)	13,364 (33.1)	18,356 (30.2)	8648 (38.4)	92,512 (31.2)	69,642 (33.9)	847,889 (27.6)	1,206,706 (28.9)
35–39	26,352 (28.0)	58,812 (15.5)	9619 (23.8)	10,245 (16.8)	6228 (27.6)	37,470 (12.6)	41,461 (20.2)	472,364 (15.4)	662,551 (15.9)
40–44	6177 (6.6)	11,302 (3.0)	2664 (6.6)	2798 (4.6)	1639 (7.3)	7043 (2.4)	10,912 (5.3)	109,565 (3.6)	152,100 (3.6)
45 +	390 (0.4)	580 (0.2)	173 (0.4)	185 (0.3)	110 (0.5)	508 (0.2)	1081 (0.5)	5809 (0.2)	8836 (0.2)
Missing	0 (0.0)	0 (0.0)	0 (0.0)	0 (0.0)	0 (0.0)	0 (0.0)	2 (< 0.01)	2 (< 0.01)	4 (< 0.01)
Sex									
Female	45,274 (48.1)	184,639 (48.5)	19,675 (48.7)	29,339 (48.2)	11,008 (48.9)	145,337 (48.9)	100,651 (49.0)	1,496,107 (48.7)	2,032,030 (48.7)
Male	48,884 (51.9)	195,865 (51.5)	20,738 (51.3)	31,481 (51.8)	11,526 (51.1)	151,639 (51.1)	104,771 (51.0)	1,577,662 (51.3)	2,142,566 (51.3)
Congenital anomaly									
No	92,292 (98.0)	372,235 (97.8)	39,590 (98.0)	59,289 (97.5)	22,029 (97.8)	288,759 (97.2)	200,488 (97.6)	2,991,809 (97.3)	4,066,491 (97.4)
Yes	1866 (2.0)	8269 (2.2)	823 (2.0)	1531 (2.5)	505 (2.2)	8217 (2.8)	4934 (2.4)	81,960 (2.7)	108,105 (2.6)

*IMD* Index of multiple deprivation, *SP* second parent

### Emergency admissions

**Fig. 3:**
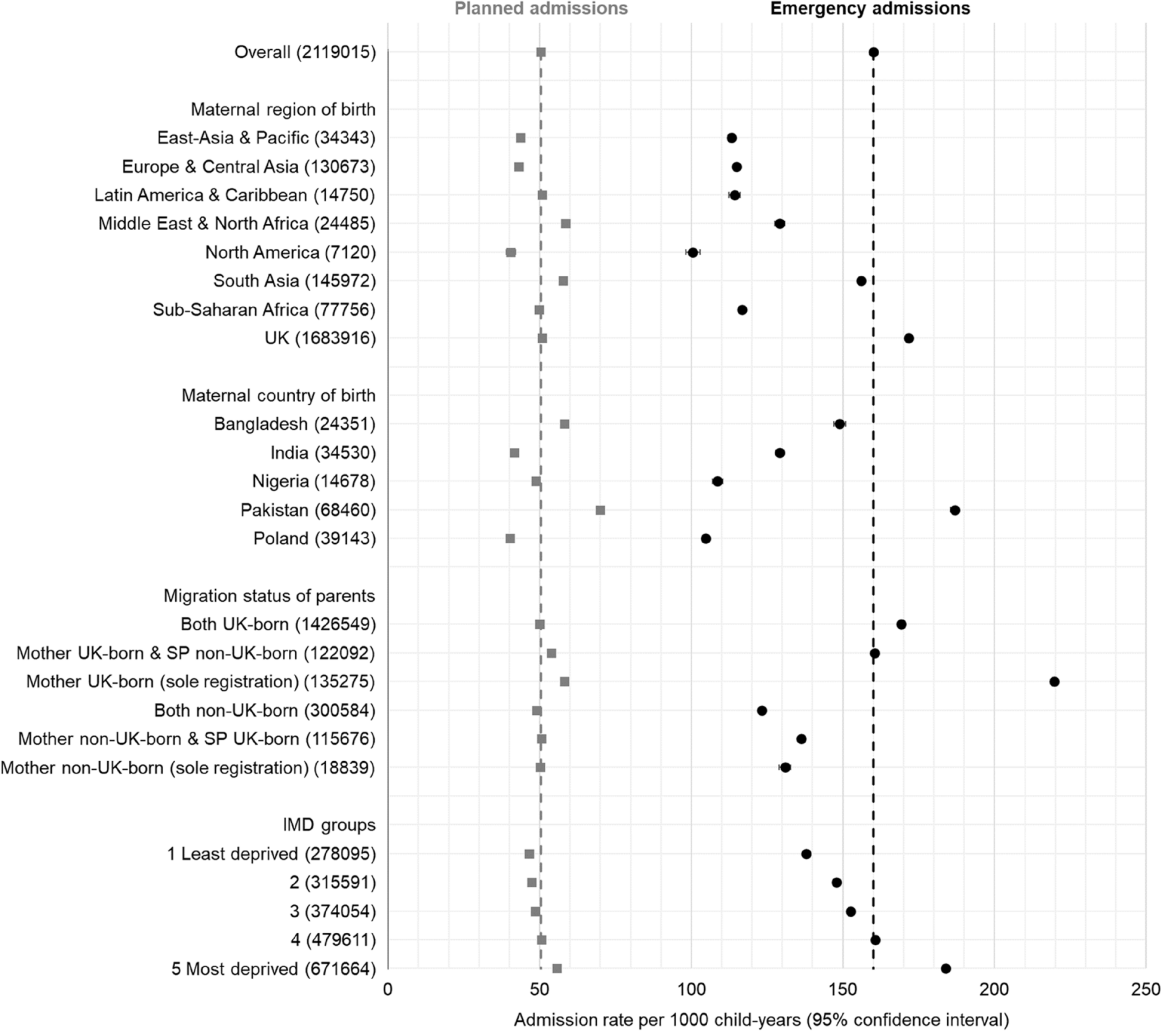
Observed incidence rates of planned and emergency hospital admissions per 1000 child-years (95% confidence intervals), by maternal world region of birth, maternal country of birth, parental migration status and IMD group; IMD = index of multiple deprivation, SP = second parent. Dashed grey and black lines indicate the cohort average observed incidence rates of planned and emergency admissions, respectively

76.0% (2,119,015/2,787,445) of hospital admissions in the cohort were emergency admissions. Within world regional groups, observed rates of emergency admissions were highest among children with UK-born mothers (171.6 per 1000 child-years, 95% CI 171.4–171.9), followed by children with mothers born in South Asia (155.9 per 1000, 95% CI 155.1–156.7) and the Middle East and North Africa (129.1 per 1000, 95% CI 127.5–130.8; Fig. 3, Additional File 6: Table S9). Children whose mothers were born in Pakistan had the highest observed emergency admission rates of the six maternal countries of birth studied (186.8 per 1000, 95% CI 185.4–188.2), followed by mothers born in the UK and Bangladesh. Children with UK-born mothers and no second parent recorded on their birth registration had the highest observed emergency admission rates of all studied groups (219.7 per 1000, 95% CI 218.5–220.9).

**Fig. 4:**
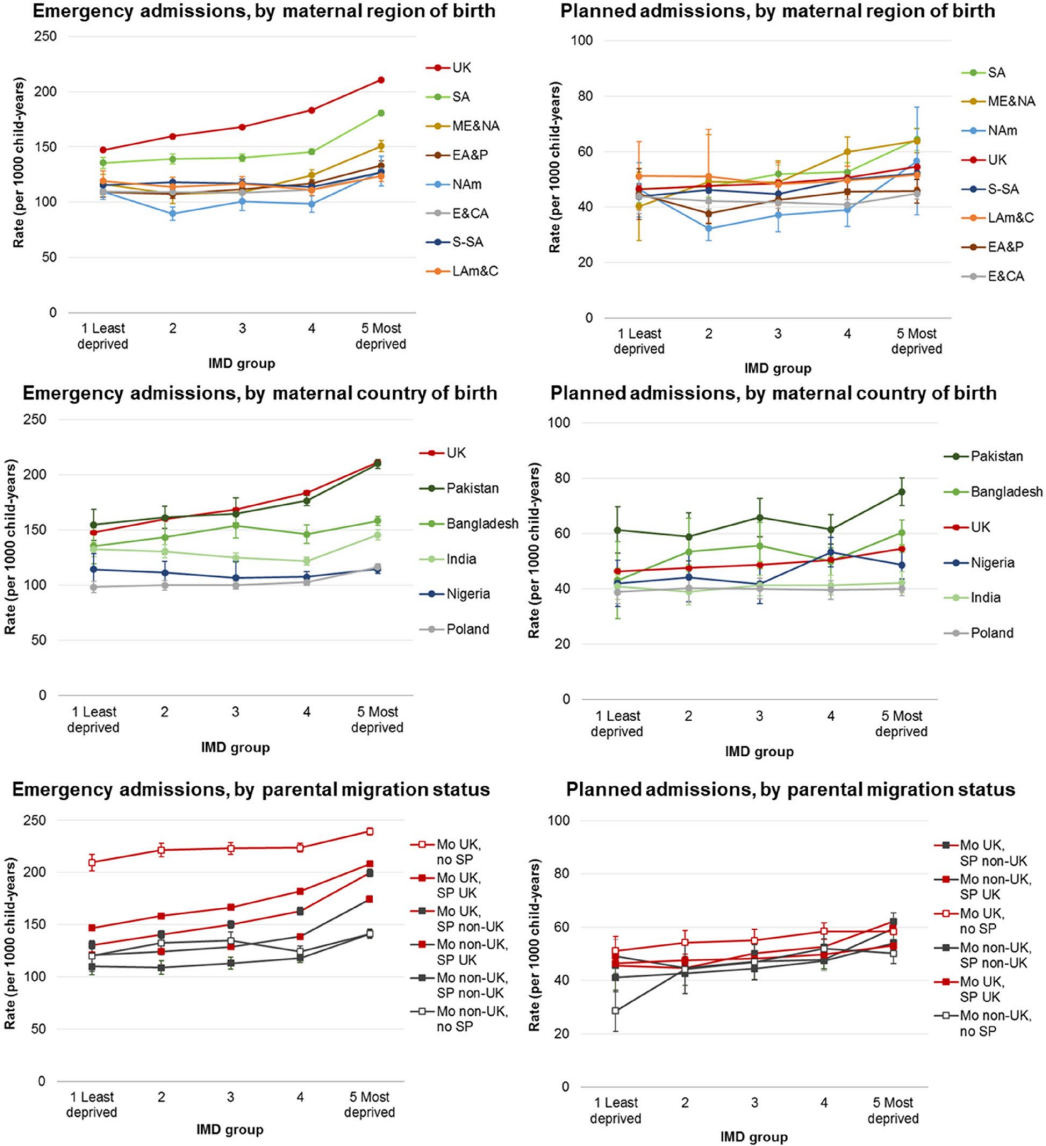
Estimated incidence rates of emergency and planned hospital admissions per 1000 child-years, by maternal world region of birth/maternal country of birth/parental migration status and IMD group (note *Y*-axis scales are different); IMD = index of multiple deprivation; EA&P = East Asia and Pacific, E&CA = Europe (excl. UK) and Central Asia, LAm&C = Latin America and Caribbean, ME&NA = Middle East and North Africa, NAm = North America, SA = South Asia, S-SA = Sub-Saharan Africa; Mo = mother, SP = second parent, UK = UK-born parent, Non-UK = non-UK-born parent

Incidence rates (estimated from negative binomial regression models) of emergency admissions rose with increasing deprivation, but were most pronounced among children with mothers born in the UK, South Asia, and the Middle East and North Africa (30–45% increased rate comparing most with least deprived IMD group; Additional File 6: Table S10, Fig. 4). Estimated rates for children with mothers born in Pakistan rose by IMD group in a pattern similar to that of children with UK-born mothers (Additional File 6: Table S11, Fig. 4). Nigeria-born mothers were the only country of birth group to display no pattern by IMD group. Among parental migrant groups, children with both parents born outside the UK had the lowest estimated rates of admissions across all IMD groups (Additional File 6: Table S12, Fig. 4).

### Planned admissions

Within maternal region of birth groups, observed rates of planned admissions were highest in children with mothers born in Middle East and North Africa (58.6 per 1000 child-years, 95% CI 57.5–59.7) and South Asia (57.7, 95% CI 57.2–58.1; Fig. 4, Additional File 6: Table S9). Children with mothers born in Pakistan and Bangladesh had observed rates of planned admissions of 70.0 per 1000 child-years (95% CI 69.2–70.9) and 58.2 per 1000 (95% CI 57.0–59.4), respectively. Within parental migration status groups, children with UK-born mothers and no second parent registered had the highest rates of planned admissions (58.2 per 1000 child-years, 95% CI 57.6–58.8). Rates of planned admissions estimated from negative binomial regression models broadly rose with increasing deprivation, but were most pronounced among children with mothers born in the Middle-East and North Africa (IRR 1.59, 95% CI 1.32–1.90, comparing most to least deprived IMD group) and South Asia (IRR 1.38, 95% CI 1.23–1.56), Additional File 6: Table S10, Fig. 4.

### Secondary outcomes

**Fig. 5:**
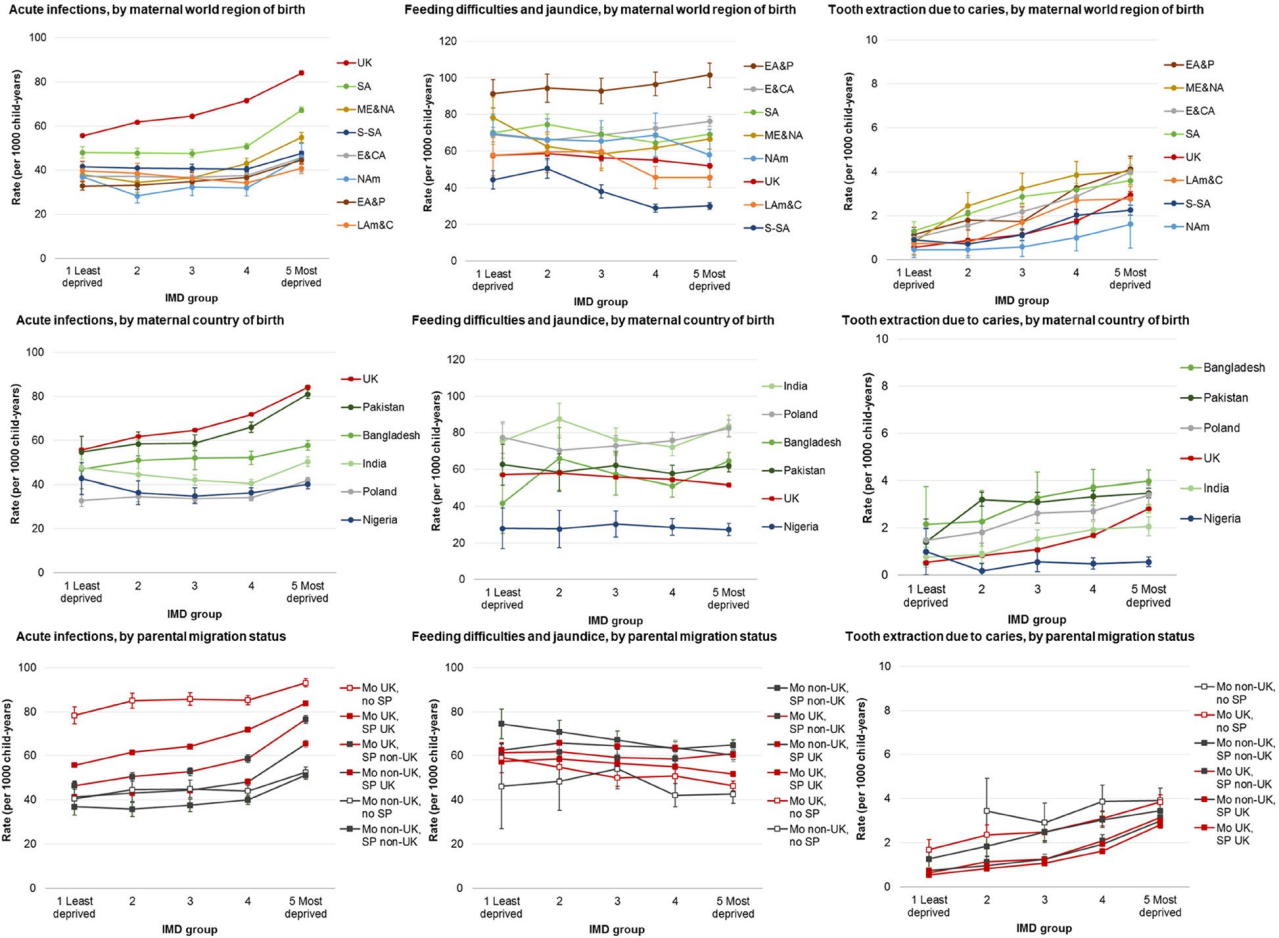
Estimated incidence rates of admissions for acute infections, feed difficulties and jaundice and tooth extractions for caries per 1000 child-years, by maternal world region of birth/maternal country of birth/parental migration status and IMD group (note *Y*-axis scales are different); IMD = index of multiple deprivation; EA&P = East Asia and Pacific, E&CA = Europe (excl. UK) and Central Asia, LAm&C = Latin America and Caribbean, ME&NA = Middle East and North Africa, NAm = North America, SA = South Asia, S-SA = Sub-Saharan Africa; Mo = mother, SP = second parent, UK = UK-born parent, Non-UK = non-UK-born parent

Acute infections comprised 37.6% of all emergency admissions in this study (795,867/2,787,445), with an overall rate of 60.1 per 1000 child-years (95% CI 60.0–60.2; Additional File 7: Tables S13). Observed and estimated rates of admissions for acute infections closely follow the patterns described for emergency admissions (Fig. 5, Additional File 7: Table S14-S16).

There were 119,742 emergency admissions for feeding difficulties and jaundice in the first 6 months of life (4.3% of all emergency admissions), a rate of 60.5 per 1000 child-years (95% CI 60.1–60.8, Additional File 7: Tables S13). Estimated rates were highest for infants of mothers born in East Asia and Pacific (and, to a lesser extent, Europe and Central Asia) in the two most deprived IMD groups (Fig. 5, Additional File 7: Table S14). Conversely, infants of mothers born in other world regions display higher estimated incidence rates in the least deprived compared to the most deprived groups.

There were 18,999 planned admissions for tooth extraction including a diagnosis of caries (2.8% of the 668,430 total planned admissions), yielding a rate of 3.10 per 1000 child-years (95% CI 3.06–3.15, Additional File 7: Table S13). These admissions were highest among children of mothers born in Middle East and North Africa (5.91 per 1000, 95% CI 5.42–6.44) and South Asia (5.31 per 1000, 95% CI 5.10–5.54). A steady pattern of increasing rates of admissions by IMD group is shown across all maternal region of birth groups, but most pronounced among children of mothers born in the UK (IRR 5.21, 95% CI 4.82–5.62) and the Midde East and North Africa (IRR 5.10, 95% CI 2.33–11.16; Fig. 5, Additional File 7: Table S14).

### Sensitivity analyses

Estimated rates of emergency and planned hospital admissions stratified by London and non-London residence at birth show similar patterns to national results presented above (Additional File 8: Figure S2). There were higher rates of emergency admissions across all maternal regions of birth and IMD groups for children living outside London compared to within London, and less differentiation between the maternal groups with the highest estimated rates (UK and South Asia) and the other world regions of birth. Results by emigration scenarios are presented in Additional File 8: Figures S3-S4. The broad patterns of emergency admission rates were similar under the various emigration projections. However, the high level of estimated rates of emergency admission among children of UK-born mothers were matched by children of South Asia-born mothers only where it is assumed that one tenth of the UK-born population with mothers born in South Asia emigrate each year.

## Discussion

We used a national birth cohort dataset from England, including data from over four million children, to examine admission rates among parental migration and socioeconomic groups. We found that overall, whilst children whose parents are born abroad were less likely to be admitted to hospital in an emergency compared to children whose parents were born in the UK, there were substantial differences by maternal place of birth and reason for admission. We also observed differences within the UK-born group, with children of mothers born in the UK, those with no second parent registered at birth having particularly high rates of emergency admissions. A socioeconomic gradient in emergency and planned admissions were observed and was most pronounced for children of mothers born in the UK, South Asia, and the Middle East and North Africa. The high rates of emergency and planned admission estimated in the South Asia group were driven by children of women born in Pakistan and, to a lesser extent, Bangladesh. Rates of admissions for feeding difficulties and jaundice were highest for infants of East Asia and Pacific-born mothers in the two most deprived socioeconomic groups, but highest in the least deprived groups among infants with mothers from Sub-Saharan Africa, Latin America and the Caribbean, and the UK. Admissions for tooth extractions due to caries displayed the greatest socioeconomic gradient, which was most pronounced among children of mothers born in the UK and the Middle East and North Africa.

### Strengths and limitations

This is the first national UK-based study to examine hospital admission rates according to parental migration in England. Linkage between birth registration and hospital admission datasets allowed us to look at parental place of birth, which is not routinely collected in NHS data, and is a novel aspect of this research. Our results offer a starting point for further investigations into health inequities experienced by specific migrant communities, with the differences in reported results by maternal place of birth highlighting the importance of a nuanced approach to this work. Nonetheless, the geographical regions (and even countries) of births used in this study comprise mixed groups of migrant women with diverse backgrounds and experiences of healthcare [34]. Unmeasured sources of heterogeneity among our non-UK born sample includes the length of time since migration, reason for migration, pre-migration socioeconomic circumstances and English proficiency [32, 35]. Only area-level measures of socioeconomic position were available in our administrative data sources, meaning that we are likely to have underestimated the true family-level effect of this determinant of health [36]. IMD scores are weighted towards the income and employment domains, which are constructed using data on benefits, meaning that IMD as a whole may be less reliable as an indicator of disadvantage among migrant groups (due to lower take up of benefits, on average) [37, 38]. Replication of this analyses with socioeconomic indicators measured at the family level, such as household income or parental education, may help to improve estimation.

Health inequity research using country of birth is well established in other European countries, with cited benefits including the objectivity and stability of this measure over time [39]. In birth registrations (used in this study), country of birth is self-reported by parents and can be harmonised using international standards. In contrast, ethnicity, which is commonly used in health inequity research in the UK, is affected by measurement error, limited response options and completeness in administrative data sources [6, 40]. Whilst documentation for HES states that ethnicity should be self-classified, it is reported that ethnicity is sometimes assigned by health care staff or, in many cases, not asked at all [41, 42]. Whilst there is a degree of overlap between geographical place of birth and ethnicity in terms of culture, language and religion, these are not the same entities. Country of origin contextualises pre-migration circumstances including migration drivers (e.g. conflict, environmental, labour, colonial migration networks), health and social care access levels, departure circumstances and wider country-level epidemiological patterns [1]. All of these factors can impact an individual’s inclusion and exclusion levels, journey and destination circumstances, and exposures and protections whilst living in England. Notably, the results of this study do not account for parents born in the UK (and elsewhere) who are from racially minoritised groups. Surveys, such as Understanding Society or the ONS Longitudinal Study, could be used to incorporate important contextual and demographic factors into future research to further understand how multiple identities intersect to impact health and healthcare use among young children.

There was no missing information for maternal region of birth in this study, showing the value of the linked birth registration records. However, 8.1% of birth registrations were excluded from this study as they did not link to an NHS record (due to a mixture of linkage error and unavailability of HES records for linkage). Linkage rates improved over study years, highlighting the value of replicating this research in new linkages of these datasets (beyond 31st December 2014; the latest date of follow-up available in this research). This study highlights the importance of linking information on parents’ country of birth from vital statistics records with children’s longitudinal health records, and several funding initiatives are aiming to make timely access to linked administrative England for research easier [43]. This reproduction is particularly important within the context of increasingly restrictive access to the NHS (and, more broadly, increasing levels of hostility and xenophobia) experienced by many migrants living in England, particularly following the UK’s Immigration Act of 2014 and 2016 and withdrawal from the European Union in 2020 [44].

### Interpretation and implications

Unlike patterns reported in other high-income countries, [5] we found that emergency admission rates were generally higher for children of UK-born parents compared with non-UK born parents, even at the same or higher levels of socioeconomic deprivation. This finding matches results of a study of linked hospital-general practice registration records in England, which estimated that recent migrants to England (indicated by first registration with a general practice after the age of 15 years) had about half the rate of hospital admissions compared with the general population of over 15 year olds [45]. Whilst we were unable to look at accident and emergency department attendance rates in this study (the most common route to emergency hospital admission) [31], there is evidence to suggest that these are also lower among children of non-UK born mothers. Using hospital data linked to the Born in Bradford cohort study, Credé et al. estimated that the odds of accident and emergency use in the first 5 years of life was 0.92 times lower among children of mothers from UK/Ireland compared to children of mothers from other countries (OR 0.92, 95% CI 0.83, 1.00) [32]. Collectively, these findings help to dispel a common (but unevidenced) perception that migrants and their young children have been placing excessive strain on the UK NHS [46].

Contrasting explanations can be posited for this major finding: that these parents and their young children are healthier and therefore require less healthcare interventions (the “healthy migrant paradox”); [47] or that they face barriers in accessing timely and preventative health services, including emergency care where needed [32]. A third explanation, of returning home for hospital treatment, as has been reported by migrants from Eastern Europe, [48] may alternatively explain particularly low admission rates for some groups of children. The healthy migrant paradox is the phenomenon, whereby, newly arrived immigrants show similar or better health (for some outcomes) than the non-migrant population despite higher levels of socioeconomic deprivation [47]. Factors thought to contribute to this phenomenon include the selective migration of younger and healthier people and resilience built through social support networks in host countries, potentially countering effects of racism and xenophobia. Factors that may contribute to lower child hospital admissions in specific communities, and warrant further investigation, include the buffering effects of living in areas with other migrants and minoritised groups who may provide support and signposting to preventive care, [49] and the role of culturally specific service provision (a factor that emerged from patient and public involvement undertaken to inform this study). Importantly, the health advantage of migrants has been shown to dissipate for most groups over time and, [47] whilst we were unable to account for time since migration in this study, Credé et al. showed higher rates of emergency department visits among children of non-UK/Ireland-born mothers who had been in the UK for 5 years or more, compared to less than 5 years [32].

Beyond the broad finding of lower emergency admissions, we find divergent patterns of hospital admissions by maternal regions/countries of birth, which suggest a more complex pattern of secondary healthcare use by geographical origin of birth. This reflects quantitative and qualitative studies of the perinatal period, which report mixed experiences of healthcare and child health outcomes in the UK across migrant women [10, 11]. Evidence of poorer early childhood health for children of mothers born in South Asia, particularly Pakistan, including increased risk of low birthweight, preterm birth and congenital anomalies, [50, 51] fits with the relatively high emergency and planned hospital admission rates shown here. However, poor early life outcomes are also reported in the UK for women born in Africa, including high rates of infant mortality, [52] sitting in contrast to the relatively low emergency hospital admission rates for children of mothers born in Sub-Saharan Africa in this study. This is particularly striking given that almost half of children with mothers born in Sub-Saharan Africa were in the most deprived socioeconomic group. This finding may represent lower levels of underlying health needs on average, perhaps owing to unmeasured differences in this population (such as the reason for migration or length of time in the UK), but could also indicate obstacles to healthcare, and warrants further investigation. Healthcare barriers specific to some groups may include less culturally and linguistically appropriate services; reduced awareness of healthcare entitlements; or, particularly in vulnerable groups, a fear of charging, or immigration enforcement [53, 54]. Linkage between birth registration and/or Census data to primary care and accident and emergency attendances, as well as outpatient datasets, and qualitative studies within diverse groups of parents who have migrated to the UK will help to elucidate the extent to which health services are meeting the underlying needs of children. Future research could focus on children with specific conditions, such as asthma or epilepsy, to capture differences in care pathways across children from different backgrounds.

A socioeconomic gradient in the rates of hospital admissions in England, as shown in this study, has been repeatedly documented in the general childhood population [15, 16]. Emergency hospital use is associated with poorer access to primary care and preventative services, and rates of both emergency and planned admissions are higher among children with chronic conditions (the prevalence of which are also driven by inequities) [16]. Planned admissions for tooth extractions for caries clearly reflects substantial oral health inequalities, [55] whereas admissions for feeding difficulties and jaundice illustrate a more complex socioeconomic pattern of secondary healthcare use. Exclusive breastfeeding (which is linked to the onset of feeding difficulties and physiological jaundice) is more common among women from affluent backgrounds in England, which likely explains the reverse social gradient shown for these admissions [26]. However, the reverse pattern among mothers born in East Asia and Pacific highlights that this inference may not be generalisable to all women.

On the whole, this evidence suggests that a continued focus on improving access to preventative health services, including antenatal, primary care and dental services, in poorer areas is necessary for all children, but with consideration of the diverse needs of parents and children from different migrant backgrounds. Support for children whose births are not jointly registered (a known vulnerable group of families) is also warranted given the high rates of admissions across all socioeconomic strata of this group [56]. In addition, strengthening public health programmes such as diet, overcrowding and environmental tobacco smoke is a complementary pathway to improving the health of all children. This should be a focus for Integrated Care Systems and the renewal of the Healthy Child Programme in England [57].

## Conclusions

This research indicates that children whose parents who have migrated to the UK generally have lower overall usage of NHS emergency secondary care services than children of UK-born parents. Our study revealed a socioeconomically graded pattern of hospital admissions for all children born in England, which were highest among those with mothers born in the UK, South Asia, and the Middle East and North Africa. Future research using linked primary and secondary care data is needed to elucidate where the UK healthcare system could better meet the needs of all children.

## Supplementary Information

Additional file 1. Defining variables (Tables S1-S4). Table S1—Data sources and variables. Table S2—Code list for secondary study outcomes. Table S3—Definitions of variables. Table S4—Code list for congenital anomalies.

Additional file 2. Figure S1—Conceptual framework.

Additional file 3. Estimating emigration (including Tables S5-S6). Table S5—assumed levels of emigration. Table S6—method to apply emigration projections.

Additional file 4. Table S7—Characteristics of unlinked birth registration records.

Additional file 5. Table S8—Five largest maternal countries of birth within each world region.

Additional file 6. Emergency and planned hospital admission results (Tables S9-S12). Table S9—Observed rates. Table S10—Estimated rates by maternal region of birth. Table S11 – Estimated rates by maternal country of birth. Table S12 – Estimated rates by parental migration status.

Additional file 7. Secondary outcome results (Tables S13-S16). Table S13 – Observed rates. Table S14—Estimated rates by maternal region of birth. Table S15 – Estimated rates by maternal country of birth. Table S16 – Estimated rates by parental migration status.

Additional file 8. Sensitivity analyses results (Figures S2-S4). Fig S2—Estimated rates of emergency admissions stratified by London/non-London residence at birth. Fig S3—Estimated rates of emergency admissions stratified by emigration sensitivity analysis scenario. Fig S4—Estimated rates of planned admissions stratified by emigration sensitivity analysis scenario.

## Data Availability

The authors do not have permission to supply data or identifiable information to third parties. Anyone wishing to access the linked datasets for research purposes should apply via the CAG to the Health Research Authority to access patient-identifiable data without consent and then to the ONS and NHS Digital. In the first instance, enquiries about access to the data analysed here should be addressed to the corresponding author and enquiries about the City Birth Cohort should be addressed to Alison Macfarlane (A.J.Macfarlane@city.ac.uk).

## Abbreviations

AIC: Akaike’s information criterion value
HES: Hospital Episode Statistics
ICD-10: International Classification of Diseases 10th Revision
IMD: Index of multiple deprivation
IRR: Incidence rate ratios
LSOA: Lower layer Super Output Area
NHS: National Health Service
ONS: Office for National Statistics
OPCS-4: Office of Population Censuses and Surveys
UK: United Kingdom
