# Supplementary material for: Parental migration, socioeconomic deprivation and hospital admissions in preschool children in England: national birth cohort study, 2008 to 2014
Source: BMC Med. 2024 Sep 27;22:416. doi: 10.1186/s12916-024-03619-1 (PMC11438240; doi:10.1186/s12916-024-03619-1)
Supplement: Supplementary file 1 — Additional file 1. Defining variables (Tables S1-S4). Table S1—Data sources and variables. Table S2—Code list for secondary study outcomes. Table S3—Definitions of variables. Table S4—Code list for congenital anomalies. [file 12916_2024_3619_MOESM1_ESM.docx]

## Additional File 1: Tables S1-S4

**Table S1. Data sources and variables used in this study**

| **Dataset** | **Collected by** | **Dataset details** | **Variables used in this study** |
| --- | --- | --- | --- |
| Birth registrations | ONS | Derived from the civil registration of live births and stillbirths, a legal requirement in England and Wales. Live births are legally required to be registered with the General Register Office within 42 days. | Child: IMD, sex, multiple birth, maternity unit, registration status.  Mother: age, country of birth  Second parent*: country of birth |
| Death registrations | ONS | A register of all deaths in England and Wales. The legal requirement for registering deaths with the General Register Office is 5 days after the date of death. However, delays can (and are legally allowed to) occur where the death has been referred to the coroner/for inquest. | Child: Date of death, cause of death |
| Birth notifications | NHS | Data recorded at the notification of the birth within 36 hours to the NHS by the attending midwife  or other birth attendant | *None (dataset used for linkage between birth registrations and HES APC delivery records)* |
| HES APC records | NHS | HES is an administrative database of English NHS-funded hospital contacts, collated by NHS Digital for the primary purpose of hospital reimbursement. HES APC records consist of all inpatient admissions | Child: Lower layer Super Output Area, date of admission, date of discharge, diagnoses, operations, sex, admission method (emergency or planned) |
| HES APC records (infant birth and maternal deliveries) | NHS | Each birth in HES APC generates at least one birth (belonging to the baby) and maternal (belonging to the mother) record, each with an attached baby or maternity “tail” that includes additional information such as birth weight, gestational age and delivery method. | Infant: Lower layer Super Output Area (to derive IMD where missing), date of birth admission (to proxy date of birth)  Mother: Ethnicity, Lower layer Super Output Area (to derive IMD where missing) |

HES APC = Hospital episode statistics admitted patient care, IMD = index of multiple deprivation, NHS = National Health Service, ONS = Office for National Statistics; *from 1 September 2009, same-sex female couples have been able to jointly register the birth of a child as mother and second parent (comprising 0.1% of all registered births in 2014)

Table S2. Code list for secondary study outcomes (primary diagnoses and emergency admissions unless specified)

| Condition(s) | ICD-10 name | ICD-10 code |
| --- | --- | --- |
| Acute infections - lower respiratory tract infections | | |
| Influenza, pneumonia | Influenza with pneumonia, virus identified  Influenza with pneumonia, virus not identified  Influenza with other respiratory manifestations, virus not identified  Viral pneumonia, not elsewhere classified  Pneumonia due to Streptococcus pneumoniae  Pneumonia due to Haemophilus influenzae  Bacterial pneumonia, not elsewhere classified  Chlamydial pneumonia  Pneumonia due to infectious organisms not elsewhere classified  Bronchopneumonia, unspecified  Lobar pneumonia, unspecified  Other pneumonia, organism unspecified  Pneumonia, unspecified | J10·0  J11·0  J11·1  J12  J13·X  J14  J15  J16·0  J16·8  J18·0  J18·1  J18·8  J18·9 |
| Bronchiolitis | Acute bronchiolitis | J21 |
|  | Unspecified acute lower respiratory infection | J22 |
| Acute infections - upper respiratory tract infections | | |
| Otitis media | Suppurative and unspecified otitis media  Otitis media in diseases classified elsewhere | H66  H67 |
| Throat infections | Acute pharyngitis  Acute tonsillitis  Acute laryngitis  Acute upper respiratory infections of multiple & unspecified sites  Chronic pharyngitis  Acute obstructive laryngitis [croup] and epiglottitis | J02  J03  J04·0  J06  J31·2  J05 |
| Acute infections - urinary tract infections (or pyelonephritis) | | |
| Kidney infections | Acute tubulo-interstitial nephritis  Chronic tubulo-interstitial nephritis  Tubulo-interstitial nephritis, not specified as acute or chronic  Pyonephrosis  Renal tubulo-interstitial disease, unspecified | N10  N11  N12  N13·6  N15·9 |
| Cystitis (bladder inflammation) | Acute cystitis  Other cystitis  Cystitis, unspecified | N30·0  N30·8  N30·9 |
|  | Urinary tract infection, site not specified | N39·0 |
| Dehydration and gastroenteritis | | |
|  | Volume depletion  Allergic and dietetic gastroenteritis and colitis  Other specified noninfective gastroenteritis and colitis  Noninfective gastroenteritis and colitis, unspecified  Salmonella enteritis  Other bacterial intestinal infections  Bacterial foodborne intoxication, unspecified  Cryptosporidiosis  Viral and other specified intestinal infections  Other gastroenteritis and colitis of infectious & unspecified origin  Gastroenteritis and colitis due to radiation  Toxic gastroenteritis and colitis | E86  K52·2  K52·8  K52·9  A02·0  A04  A05·9  A07·2  A08  A09  K52·0  K52·1 |
| Other/unknown |  |  |
|  | Viral infection of unspecified site | B34 |
| Feeding difficulties and jaundice | | |
|  | Neonatal jaundice from other and unspecified causes  Feeding problems of new-born | P59  P92 |
| Tooth extractions for caries* (planned admissions only) | | |
| Dental caries | Caries of dentine  Dental caries on pit and fissure surface  Other dental caries  Dental caries, unspecified  Diseases of pulp and periapical tissues  Chronic apical periodontitis  Periapical abscess with sinus  Periapical abscess without sinus | K02·1  K02·5  K02·8  K02·9  K04·0  K04·5  K04·6  K04·7 |
| Tooth extraction | Surgical removal of tooth (main operative procedure)  Simple extraction of tooth (main operative procedure) | F09 (OPCS code)  F10 (OPCS code) |

ICD-10 = International Classification of Diseases and Related Health Problems 10th Revision; OPCS = Office of Population Census and Survey codes (surgical procedure codes); *To meet definition, admission must include a primary diagnosis of dental caries AND one of the two listed operative procedures

**Table S3. Definitions of exposures, outcomes and covariates**

| **Variable type** | **Variable name** | **Definition/values** | **Origin dataset** |
| --- | --- | --- | --- |
| Exposure | Maternal region of birth | Eight groups, adapted from the World Bank’s 7-group classification of geographical regions: East-Asia & Pacific Europe (excl. UK) & Central Asia; Latin America & Caribbean; Middle East & North Africa; North America; South Asia; Sub-Saharan Africa; UK | Birth registrations |
| Exposure | Maternal country of birth | Six most common countries in our dataset: Bangladesh; India; Nigeria; Pakistan; Poland; the UK | Birth registrations |
| Exposure | Parental migration status | Both UK-born; Mother UK-born & SP non-UK-born; Mother UK-born (sole registration); Both non-UK-born; Mother non-UK-born; SP UK-born  Mother non-UK-born (sole registration) | Birth registrations |
| Outcome | Planned admissions | Hospital admission with a planned admission method | HES APC (child post-birth admissions) |
| Outcome | Emergency admissions | Hospital admission with an emergency admission method | HES APC (child post-birth admissions) |
| Outcome | Acute infections | Emergency hospital admission with a diagnosis of: lower respiratory tract infection; upper respiratory tract infection; urinary tract infection, dehydration; or gastroenteritis (see Supplementary Table 2) | HES APC (child post-birth admissions) |
| Outcome | Neonatal feeding difficulties | Emergency hospital admission with a diagnosis of: neonatal jaundice from other and unspecified causes; or feeding problems of new-born (see Supplementary Table 2) | HES APC (child post-birth admissions) |
| Outcome | Tooth extractions due to caries | Planned hospital admissions with a diagnosis of: Dental caries; and a main operative procedure of surgical removal of tooth, or simple extraction of tooth (see Supplementary Table 2) | HES APC (child post-birth admissions) |
| Covariate | IMD | IMD 2010 split into quintiles from most to least deprived | ONS birth registration (supplemented by HES APC infant birth or maternal delivery records if missing) |
| Covariate | Year of birth | 2008 to 2014 | Birth registrations |
| Covariate* | Child sex | Physician assigned sex at birth: Female or male | Birth registrations |
| Covariate* | Geographical region of residence | London, the North East, North West, Yorkshire and the Humber, East Midlands, West Midlands, South East, East of England, the South West | Birth registrations |
| Covariate* | Maternal ethnicity | HES coded ethnicity (guidance stipulates self-reporting, but cases of staff-assigned ethnicity have been documented): Bangladeshi, Indian, Pakistani, Black African, Black Caribbean, White British, White Other, Other, Not known or missing | HES APC (maternal delivery record) |
| Covariate* | Maternal age | maternal age at the birth of the child (split into <20, 20-29, 30-39, 40+ years) | Birth registrations |
| Covariate* | Congenital anomalies | Presence of congenital anomalies (yes or no), based on the Hardelid UK chronic condition ICD-10 code list,[^4^](https://www.zotero.org/google-docs/?SpYirz) were identified in child hospital admissions or death certificates up to age two years. See supplementary table 4 | HES APC (child post-birth admissions) |

HES APC = hospital episode statistics admitted patient care, IMD = Index of Multiple Deprivation, SP = second parent; *Covariate included to present information on cohort sociodemographic and health characteristics only

Table S4. ICD-10 codes used to define congenital anomalies

| ICD-10 subchapter | ICD-10 codes |
| --- | --- |
| Congenital malformation of the nervous system | Q00 Q01 Q02 Q03 Q04 Q05 Q06 Q07 |
| Congenital malformations of eye, ear, face and neck | Q104 Q107 Q11 Q12 Q130-Q134 Q138 Q139 Q14 Q15 Q16 Q188 |
| Congenital malformations of the circulatory system | Q20 Q21 Q22 Q23 Q24 Q25 Q26 Q27 Q28 |
| Congenital malformations of the respiratory system | Q30 Q31 Q32 Q33 Q34 |
| Cleft lip and cleft palate | Q35 Q36 Q37 |
| Other congenital malformations of the digestive system | Q380 Q383 Q384 Q386-Q388 Q39 Q402-Q409 Q41 Q42 Q431 Q433-Q437 Q439 Q44 Q45 |
| Congenital malformations of genital organs | Q500 Q51 Q520-Q522 Q524 Q540-Q543 Q548 Q549 Q550 Q555 Q56 |
| Congenital malformations of the urinary system | Q601 Q602 Q604-Q606 Q61 Q620-Q626 Q628 Q630-Q632 Q638 Q639 Q64 |
| Congenital malformations and deformations of the musculoskeletal system | Q650-Q652 Q658 Q659 Q675 Q682-Q685 Q71 Q72 Q73 Q74 Q750 Q751 Q753-Q759 Q761-Q764 Q77 Q78 Q790 Q792-Q798 |
| Other congenital malformations | Q80 Q81 Q820-Q824 Q829 Q85 Q86 Q87 Q891-Q899 |
| Chromosomal abnormalities not elsewhere classified | Q90 Q91 Q92 Q93 Q952-Q953 Q97 Q980 Q99 |

ICD-10 = International Classification of Diseases and Related Health Problems 10th Revision
