# Supplementary material for: Parental migration, socioeconomic deprivation and hospital admissions in preschool children in England: national birth cohort study, 2008 to 2014
Source: BMC Med. 2024 Sep 27;22:416. doi: 10.1186/s12916-024-03619-1 (PMC11438240; doi:10.1186/s12916-024-03619-1)
Supplement: Supplementary file 2 — Additional file 2. Figure S1—Conceptual framework. [file 12916_2024_3619_MOESM2_ESM.docx]

##
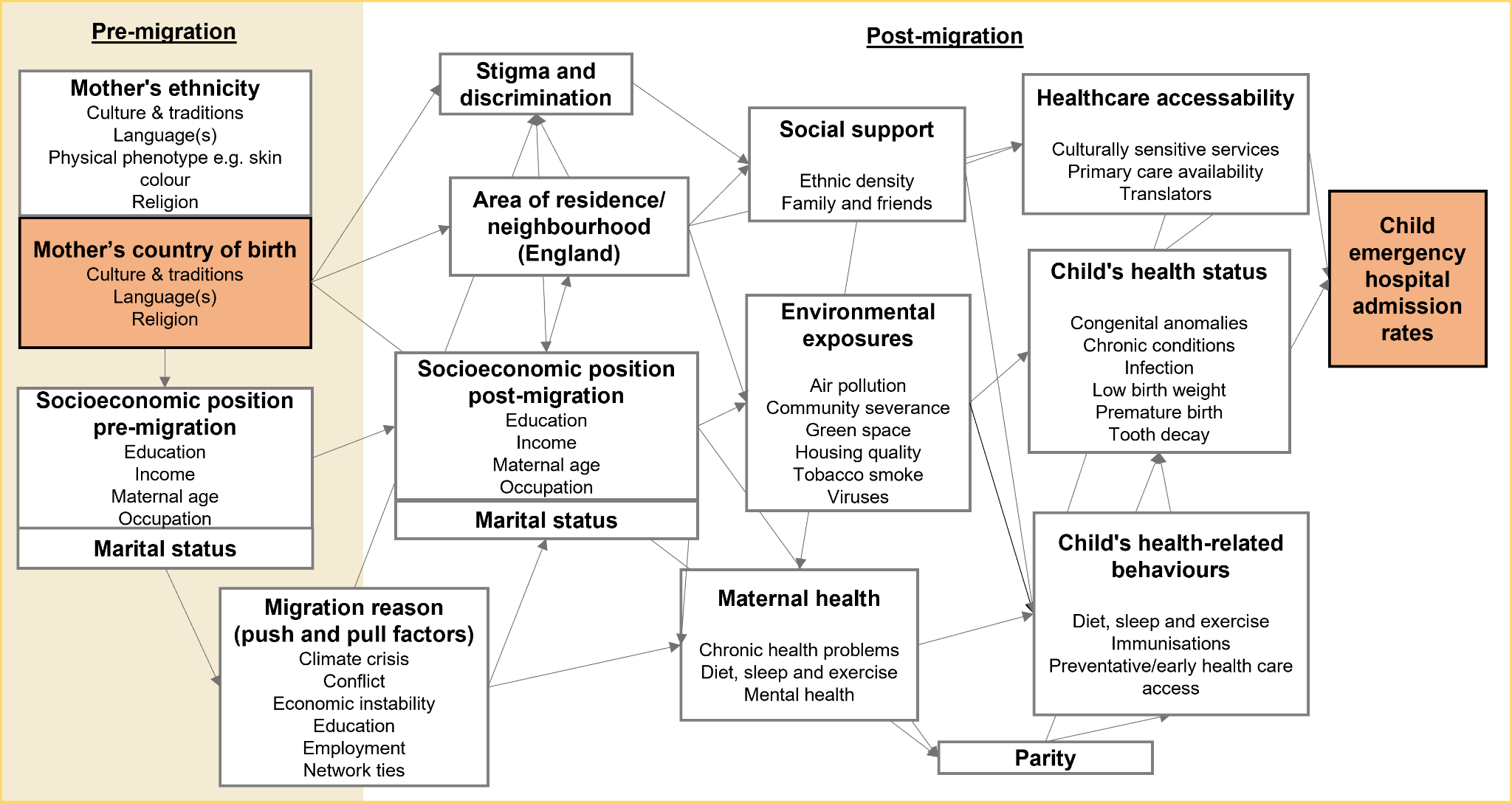
Additional File 2: FigS1

Figure S1. Conceptual framework showing potential pathways between maternal country of birth and offspring emergency hospital admissions rates; orange box indicates exposure or outcome
