# Supplementary material for: Parental migration, socioeconomic deprivation and hospital admissions in preschool children in England: national birth cohort study, 2008 to 2014
Source: BMC Med. 2024 Sep 27;22:416. doi: 10.1186/s12916-024-03619-1 (PMC11438240; doi:10.1186/s12916-024-03619-1)
Supplement: Supplementary file 3 — Additional file 3. Estimating emigration (including Tables S5-S6). Table S5—assumed levels of emigration. Table S6—method to apply emigration projections. [file 12916_2024_3619_MOESM3_ESM.docx]

## Additional File 3: Sensitivity analyses - estimating emigration in the national birth cohort

### Outlining the problem

Our study datasets do not include information on emigration of the children in the cohort during follow up. Not including this information may bias the results by overestimating the denominator (i.e. the child-years) and therefore underestimating rates of admissions among emigrants. Given that some populations are more likely to be mobile, the impact of this bias is unlikely to be uniform across maternal world region of birth or parental migration status groups. It is therefore important to carry out some sensitivity analyses assuming different scenarios of emigration to assess how this bias may affect the results of our study. We do not need to measure immigration or net migration for our work because our cohort includes all children born in England to resident women.

### Suggested solutions

The ideal solution to our problem would be individual-level linkage to records of emigration. This is not currently possible. We are due to get embarkation dates from the Personal Demographics Service (PDS) for the children in the cohort from NHS Digital. The PDS will include records of anyone who has let their GP or other NHS care provider know they are moving out of England. However, these data are likely to be highly incomplete, as not all those who emigrate alert their GP. The second best option is to impute probable rates of emigration based on aggregate information of rates of emigration, matched on key variables such as maternal age and region of birth. This requires a dataset(s) with a reliable measure of the size (or stock) of the population at risk of migration and emigration rates over time.

### Official statistics

Estimating migration stock: the Annual Population Survey (APS) provides an estimate of the number of people living in private households in the UK, by geographical area and key characteristics (including country of birth).[^7^](https://www.zotero.org/google-docs/?G1pQGL) The APS is created by combining 2 waves of the main Labour Force Survey (a continuous household survey of the UK) with data collected on a local sample boost.

Estimating migration flows: The official source of data on immigration and emigration, produced by the Office for National Statistics (ONS), is the Long-Term International Migration (LTIM) series (previously called the Total International Migration statistics or TIM).[^8^](https://www.zotero.org/google-docs/?0AZiTP) LTIM is primarily created using information from the International Passenger Survey (IPS), which is a sample survey carried out at all main ports in the UK to capture migration intentions.[^9^](https://www.zotero.org/google-docs/?M0yOr6) Owing to some methodological problems with the IPS (including sampling problems and uncertain intentions by survey respondents), the data since 2008 has been adjusted using other data sources. Since 2019, the LTIM is classified as experimental statistics whilst further work is being undertaken to improve this dataset.

### Conclusion from available data

Theoretically it would be possible to combine data from LTIM and the APS to calculate migration rates according to sex, age and region of birth, which would allow us to carry out the above imputation analyses. However, the ONS has specifically written that the LTIM and APS dataset should not be used in conjunction with one another to estimate the proportion of the population who migrate.^5^ As such, there is no one single source of data in the UK that can be used to reliably model the proportion of the population that emigrates over a period of time. In any case, neither the LTIM or the APS have data specifically on the emigration of women who have given birth in the last five years, nor migration of children aged less than five years old according to their parents’ country of birth. The ONS is in the process of transforming population and migration statistics by using a combination of administrative data sources and has written about producing triangulated population stock and flow data by population characteristics.[^10^](https://www.zotero.org/google-docs/?E81dMv) But this information is not yet available, and may not cover our study years (2008 to 2014) in any case.

### Working solution

In the absence of reliable data on emigration, we will therefore model four simple scenarios, each with different levels of assumed annual emigration by maternal world region of birth (as shown in Supplementary Table 5). The first two scenarios consider all groups apart from the UK-born group to have the same (higher) levels of emigration. The last two scenarios consider higher levels of emigration among children with mothers born in East-Asia and Pacific, Europe (excluding the UK) & Central Asia, and North America. This differentiation is based on a Home Office report on patterns of long term emigration in the UK up to 2011, which concludes that people from higher-income non-UK countries (including EU countries, New-Zealand and Australia) are less likely to stay permanently in the UK.[^11^](https://www.zotero.org/google-docs/?IEmbLI)

We make several additional assumptions when doing these simulations

1. For each age group each year a uniform percentage of children emigrate
2. Emigration implies not returning to England during follow up time
3. Children who migrate do so half-way through the year of follow up

Supplementary table 6 outlines how the data were transformed in each emigration scenario. Following this process, the data were analysed according to the methods described in the main paper (for maternal region of birth as the exposure and emergency admissions rates as the outcome).

Table S5. Assumed levels of emigration (*x* in Table S6) for each scenario, by maternal world region of birth

|  | Scenario 1 | Scenario 2 | Scenario 3 | Scenario 4 |
| --- | --- | --- | --- | --- |
| East-Asia & Pacific | 5% | 10% | 5% | 10% |
| Europe (excl. UK) & Central Asia | 5% | 10% | 5% | 10% |
| Latin America & Caribbean | 5% | 10% | 1% | 1% |
| Middle East & North Africa | 5% | 10% | 1% | 1% |
| North America | 5% | 10% | 5% | 10% |
| South Asia | 5% | 10% | 1% | 1% |
| Sub-Saharan Africa | 5% | 10% | 1% | 1% |
| UK | 1% | 1% | 1% | 1% |

Table S6. Method to apply emigration projections within maternal region of birth groups

| Year | Child age (years) | Method (where *x* = % specified in Table S5) |
| --- | --- | --- |
| 2008 | 0 | Set end of follow up to 0.5 years for random x% of cohort members (only among those with no admissions in follow up) |
| 2009 | 0, 1 | Set end of follow up to 0.5 (for those born in 2009) or 1.5 (for those born in 2008) years for random x% of cohort members within each birth year (among those with no cases between 2009 and 2014) |
| 2010 | 0, 1, 2 | Set end of follow up to 0.5 (for those born in 2010), 1.5 (for those born in 2009) or 2.5 (for those born in 2008) years for random x% of cohort members within each birth year (among those with no cases between 2010 and 2014) |
| 2011 | 0, 1, 2, 3 | Set end of follow up to 0.5 (for those born in 2011), 1.5 (for those born in 2010), 2.5 (for those born in 2009) or 3.5 (for those born in 2008) years for random x% of cohort members within each birth year (only those with no cases between 2011 and 2014) |
| 2012 | 0, 1, 2, 3, 4 | Set end of follow up to 0.5 (for those born in 2012), 1.5 (for those born in 2011), 2.5 (for those born in 2010), 3.5 (for those born in 2009) or 4.5 (for those born in 2008) years for random x % of cohort members within each birth year (only those with no cases between 2012 and 2014) |
| 2013 | 0, 1, 2, 3, 4 | Set end of follow up to 0.5 (for those born in 2013), 1.5 (for those born in 2012), 2.5 (for those born in 2011), 3.5 (for those born in 2010) or 4.5 (for those born in 2009) years for random x% of cohort members within each birth year (only those with no cases between 2013 and 2014) |
| 2014 | 0, 1, 2, 3, 4 | Set end of follow up to 0.5 (for those born in 2014), 1.5 (for those born in 2013), 2.5 (for those born in 2012), 3.5 (for those born in 2011) or 4.5 (for those born in 2010) years for random x% of cohort members within each birth year (only those with no cases in 2014) |
