# Supplementary material for: Parental migration, socioeconomic deprivation and hospital admissions in preschool children in England: national birth cohort study, 2008 to 2014
Source: BMC Med. 2024 Sep 27;22:416. doi: 10.1186/s12916-024-03619-1 (PMC11438240; doi:10.1186/s12916-024-03619-1)
Supplement: Supplementary file 4 — Additional file 4. Table S7—Characteristics of unlinked birth registration records. [file 12916_2024_3619_MOESM4_ESM.docx]

## Additional File 4: Table S7

**Table S7. Characteristics of unlinked ONS birth registration-HES APC records**

|  | Total births | Births (from ONS birth registration data) not linked to HES-APC records | | |
| --- | --- | --- | --- | --- |
|  | N | N | % of total | Unadjusted  OR (95% CI)* |
| Overall | 4,560,665 | 371,316 | 8.1 |  |
| Maternal region of birth |  |  |  |  |
| East-Asia & Pacific | 101,727 | 7,280 | 7.2 | 1.26 (1.23, 1.30) |
| Europe & Central Asia | 409,952 | 28,164 | 6.9 | 1.21 (1.19, 1.23) |
| Latin America & Caribbean | 44,720 | 4,110 | 9.2 | 1.66 (1.60, 1.72) |
| Middle East & North Africa | 65,460 | 4,404 | 6.7 | 1.18 (1.14, 1.22) |
| North America | 25,024 | 2,409 | 9.6 | 1.75 (1.67, 1.83) |
| South Asia | 316,490 | 18,203 | 5.8 | Ref. |
| Sub-Saharan Africa | 227,172 | 20,749 | 9.1 | 1.65 (1.61, 1.68) |
| UK | 3,369,437 | 285,314 | 8.5 | 1.52 (1.49, 1.54) |
| Missing | 683 | 100 | 14.6 |  |
| Migration status of parents |  |  |  |  |
| Both UK-born | 2,891,186 | 234,689 | 8.1 | 1.24 (1.23, 1.25) |
| Mother UK-born & SP non-UK-born | 261,888 | 23,431 | 8.9 | 1.38 (1.36, 1.40) |
| Mother UK-born (sole registration) | 216,363 | 27,194 | 12.6 | 2.02 (1.98, 2.05) |
| Both non-UK-born | 848,492 | 56,497 | 6.7 | Ref. |
| Mother non-UK-born & SP UK-born | 291,820 | 23,808 | 8.2 | 1.25 (1.23, 1.27) |
| Mother non-UK-born (sole registration) | 50,766 | 5,048 | 9.9 | 1.55 (1.50, 1.60) |
| Missing | 150 | 66 | 44.0 |  |
| IMD groups |  |  |  |  |
| 1 Least deprived | 636,622 | 49,641 | 7.8 | Ref. |
| 2 | 748,473 | 58,748 | 7.8 | 1.01 (0.99, 1.02) |
| 3 | 877,155 | 71,143 | 8.1 | 1.04 (1.03, 1.06) |
| 4 | 1,038,197 | 82,818 | 8.0 | 1.03 (1.01, 1.04) |
| 5 Most deprived | 1,256,984 | 107,087 | 8.5 | 1.10 (1.09, 1.11) |
| Missing | 3,234 | 1,296 | 40.1 |  |
| Year of birth |  |  |  |  |
| 2008 | 648,609 | 96,615 | 14.9 | 2.70 (2.67, 2.73) |
| 2009 | 646,192 | 61,274 | 9.5 | 1.62 (1.60, 1.64) |
| 2010 | 658,283 | 48,035 | 7.3 | 1.21 (1.20, 1.23) |
| 2011 | 659,188 | 43,560 | 6.6 | 1.09 (1.08, 1.11) |
| 2012 | 669,505 | 43,008 | 6.4 | 1.06 (1.04, 1.07) |
| 2013 | 641,217 | 39,023 | 6.1 | Ref. |
| 2014 | 637,671 | 39,218 | 6.2 | 1.01 (1.00, 1.03) |
| Region of residence |  |  |  |  |
| North East | 202,401 | 7,615 | 3.8 | Ref. |
| North West | 595,580 | 57,592 | 9.7 | 2.74 (2.67, 2.81) |
| Yorkshire & Humber | 448,229 | 29,603 | 6.6 | 1.81 (1.76, 1.86) |
| East Midlands | 368,236 | 24,946 | 6.8 | 1.86 (1.81, 1.91) |
| West Midlands | 485,901 | 44,096 | 9.1 | 2.55 (2.49, 2.62) |
| East of England | 485,977 | 41,725 | 8.6 | 2.40 (2.34, 2.46) |
| London | 865,799 | 74,329 | 8.6 | 2.40 (2.35, 2.46) |
| South East | 706,711 | 61,792 | 8.7 | 2.45 (2.39, 2.51) |
| South West | 401,610 | 28,814 | 7.2 | 1.98 (1.93, 2.03) |
| Missing | 221 | 221 | 100.0 |  |

HES APC = hospital episode statistics admitted patient care, IMD = index of multiple deprivation, OR = odds ratio, SP = second parent; *unadjusted logistic regression of the association between key characteristics and no available HES APC birth record (due to opt outs and non-linkage)
