# Supplementary material for: Parental migration, socioeconomic deprivation and hospital admissions in preschool children in England: national birth cohort study, 2008 to 2014
Source: BMC Med. 2024 Sep 27;22:416. doi: 10.1186/s12916-024-03619-1 (PMC11438240; doi:10.1186/s12916-024-03619-1)
Supplement: Supplementary file 5 — Additional file 5. Table S8—Five largest maternal countries of birth within each world region. [file 12916_2024_3619_MOESM5_ESM.docx]

## Additional File 5: Table S8

Table S8. Five largest maternal countries of birth within each world region (defined by the number of children in the birth cohort)

| East-Asia & Pacific (*N* = 94,158) | | | |  | North America (*N* = 22,534) | | | |
| --- | --- | --- | --- | --- | --- | --- | --- | --- |
|  | Country | N | % |  |  | Country | N | % |
| 1 | China | 21,416 | 22.7 |  | 1 | United States | 15,620 | 69.3 |
| 2 | Philippines | 16,672 | 17.7 |  | 2 | Canada | 6,600 | 29.3 |
| 3 | Australia | 12,583 | 13.4 |  | 3 | Bermuda | 310 | 1.4 |
| 4 | New Zealand | 6,693 | 7.1 |  | 4 | # | # | # |
| 5 | Thailand | 6,526 | 6.9 |  | 5 | # | # | # |
|  |  |  |  |  |  |  |  |  |
| Europe & Central Asia (*N* = 380,504) | | | |  | South Asia (*N* = 296,976) | | | |
|  | Country | N | % |  |  | Country | N | % |
| 1 | Poland* | 122,889 | 32.3 |  | 1 | Pakistan* | 115,886 | 39.0 |
| 2 | Germany | 29,050 | 7.6 |  | 2 | India* | 85,830 | 28.9 |
| 3 | Romania | 23,438 | 6.2 |  | 3 | Bangladesh* | 50,831 | 17.1 |
| 4 | Lithuania | 23,209 | 6.1 |  | 4 | Sri Lanka | 21,357 | 7.2 |
| 5 | Ireland | 17,503 | 4.6 |  | 5 | Afghanistan | 17,495 | 5.9 |
|  |  |  |  |  |  |  |  |  |
| Latin America & Caribbean (*N* = 42,211) | | | |  | Sub-Saharan Africa (*N* = 205,422) | | | |
|  | Country | N | % |  |  | Country | N | % |
| 1 | Jamaica | 12,728 | 31.5 |  | 1 | Nigeria* | 42,934 | 20.9 |
| 2 | Brazil | 8,984 | 22.2 |  | 2 | Somalia | 33,614 | 16.4 |
| 3 | Colombia | 3,217 | 8.0 |  | 3 | South Africa | 25,032 | 12.2 |
| 4 | Trinidad and Tobago | 1,755 | 4.3 |  | 4 | Ghana | 20,735 | 10.1 |
| 5 | Ecuador | 1,448 | 3.6 |  | 5 | Zimbabwe | 16,189 | 7.9 |
|  |  |  |  |  |  |  |  |  |
| Middle East & North Africa (*N* = 60,820) | | | |  | UK* (*N* = 3,073,769) | | | |
|  | Country | N | % |  |  | Nation/country | N | % |
| 1 | Iraq | 13,988 | 23.0 |  | 1 | England | 2,987,370 | 97.2 |
| 2 | Algeria | 5,967 | 9.8 |  | 2 | Scotland | 39,444 | 1.3 |
| 3 | Iran | 5,594 | 9.2 |  | 3 | Wales | 31,569 | 1.0 |
| 4 | Morocco | 4,603 | 7.6 |  | 4 | Northern Ireland | 13,194 | 0.4 |
| 5 | Libya | 4,519 | 7.4 |  | 5 | Jersey | 845 | <0.01 |

# Supressed due to low cell count and other values rounded to nearest 10; *Included in country of birth analyses
