# Supplementary material for: Parental migration, socioeconomic deprivation and hospital admissions in preschool children in England: national birth cohort study, 2008 to 2014
Source: BMC Med. 2024 Sep 27;22:416. doi: 10.1186/s12916-024-03619-1 (PMC11438240; doi:10.1186/s12916-024-03619-1)
Supplement: Supplementary file 6 — Additional file 6. Emergency and planned hospital admission results (Tables S9-S12). Table S9—Observed rates. Table S10—Estimated rates by maternal region of birth. Table S11 – Estimated rates by maternal country of birth. Table S12 – Estimated rates by parental migration status. [file 12916_2024_3619_MOESM6_ESM.docx]

## Additional File 6: Main results - emergency and planned hospital admissions

**Table S9.** **Observed numbers (%) and rates (per 1000 child-years) of emergency and planned hospital admissions, by maternal region of birth, maternal country of birth, migration status of parents and IMD group: England, births from 2008 to 2014**

|  | **Emergency admissions** | | **Planned admissions** | |
| --- | --- | --- | --- | --- |
|  | **N (% of cases)** | **Rate per 1000 child-years (95% CI)** | **N (% of cases)** | **Rate per 1000 child-years (95% CI)** |
| Overall | 2119015 (100.0) | 160.0 (159.8, 160.2) | 668430 (100.0) | 50.5 (50.3, 50.6) |
| **Maternal region of birth** |  |  |  |  |
| East-Asia & Pacific | 34343 (1.6) | 113.3 (112.1, 114.5) | 13228 (2.0) | 43.6 (42.9, 44.4) |
| Europe & Central Asia | 130673 (6.2) | 114.8 (114.2, 115.5) | 48957 (7.3) | 43.0 (42.6, 43.4) |
| Latin America & Caribbean | 14750 (0.7) | 114.3 (112.5, 116.2) | 6565 (1.0) | 50.9 (49.7, 52.1) |
| Middle East & North Africa | 24485 (1.2) | 129.1 (127.5, 130.8) | 11113 (1.7) | 58.6 (57.5, 59.7) |
| North America | 7120 (0.3) | 100.5 (98.2, 102.8) | 2867 (0.4) | 40.5 (39.0, 42.0) |
| South Asia | 145972 (6.9) | 155.9 (155.1, 156.7) | 53986 (8.1) | 57.7 (57.2, 58.1) |
| Sub-Saharan Africa | 77756 (3.7) | 116.7 (115.9, 117.5) | 33184 (5.0) | 49.8 (49.3, 50.3) |
| UK | 1683916 (79.5) | 171.6 (171.4, 171.9) | 498530 (74.6) | 50.8 (50.7, 51.0) |
| **Maternal country of birth** |  |  |  |  |
| Bangladesh | 24351 (1.1) | 148.9 (147.1, 150.8) | 9516 (1.4) | 58.2 (57.0, 59.4) |
| India | 34530 (1.6) | 129.2 (127.9, 130.6) | 11109 (1.7) | 41.6 (40.8, 42.4) |
| Nigeria | 14678 (0.7) | 108.6 (106.9, 110.4) | 6593 (1.0) | 48.8 (47.6, 50.0) |
| Pakistan | 68460 (3.2) | 186.8 (185.4, 188.2) | 25674 (3.8) | 70.0 (69.2, 70.9) |
| Poland | 39143 (1.8) | 104.8 (103.7, 105.8) | 15007 (2.2) | 40.2 (39.5, 40.8) |
| **Migration status of parents** |  |  |  |  |
| Both UK-born | 1426549 (67.3) | 169.1 (168.8, 169.4) | 421773 (63.1) | 50.0 (49.9, 50.2) |
| Mother UK-born & SP non-UK-born | 122092 (5.8) | 160.5 (159.6, 161.4) | 40899 (6.1) | 53.8 (53.2, 54.3) |
| Mother UK-born (sole registration) | 135275 (6.4) | 219.7 (218.5, 220.9) | 35858 (5.4) | 58.2 (57.6, 58.8) |
| Both non-UK-born | 300584 (14.2) | 123.2 (122.8, 123.6) | 119615 (17.9) | 49.0 (48.7, 49.3) |
| Mother non-UK-born & SP UK-born | 115676 (5.5) | 136.2 (135.4, 137.0) | 43070 (6.4) | 50.7 (50.2, 51.2) |
| Mother non-UK-born (sole registration) | 18839 (0.9) | 130.9 (129.0, 132.7) | 7215 (1.1) | 50.1 (49.0, 51.3) |
| **IMD groups** |  |  |  |  |
| 1 Least deprived | 278095 (13.1) | 137.8 (137.3, 138.3) | 93767 (14.0) | 46.5 (46.2, 46.8) |
| 2 | 315591 (14.9) | 147.9 (147.4, 148.5) | 101094 (15.1) | 47.4 (47.1, 47.7) |
| 3 | 374054 (17.7) | 152.5 (152.0, 152.9) | 119090 (17.8) | 48.5 (48.3, 48.8) |
| 4 | 479611 (22.6) | 160.6 (160.2, 161.1) | 151424 (22.7) | 50.7 (50.5, 51.0) |
| 5 Most deprived | 671664 (31.7) | 183.8 (183.4, 184.3) | 203055 (30.4) | 55.6 (55.3, 55.8) |

CI = Confidence interval, IMD = index of multiple deprivation, SP = second parent

Table S10. Estimated incidence rates and adjusted IRRs of emergency and planned hospital admissions, by maternal region of birth and IMD group (derived from negative binomial regression models)*

|  | Emergency admissions** | | | Planned admissions*** | | |
| --- | --- | --- | --- | --- | --- | --- |
|  | Incidence rate (95% CI)  per 1000 child-years | IRR (95% CI) | p-value | Incidence rate (95% CI)  per 1000 child-years | IRR (95% CI) | p-value |
| East-Asia & Pacific |  |  |  |  |  |  |
| 1 Least deprived | 109.2 (104.8, 113.5) | Ref. |  | 44.7 (35.6, 53.9) | Ref. |  |
| 2 | 107.5 (103.5, 111.5) | 0.99 (0.93, 1.04) | 0.59 | 37.7 (34.0, 41.4) | 0.84 (0.67, 1.06) | 0.14 |
| 3 | 111.6 (107.4, 115.8) | 1.02 (0.97, 1.08) | 0.42 | 42.6 (36.5, 48.8) | 0.95 (0.74, 1.22) | 0.70 |
| 4 | 116.8 (113.1, 120.5) | 1.07 (1.02, 1.13) | <0.01 | 45.6 (41.4, 49.8) | 1.02 (0.82, 1.27) | 0.87 |
| 5 Most deprived | 133.2 (129.1, 137.3) | 1.22 (1.16, 1.28) | <0.01 | 45.8 (41.5, 50.1) | 1.02 (0.82, 1.28) | 0.84 |
| Europe (excluding UK) & Central Asia |  |  |  |  |  |  |
| 1 Least deprived | 109.5 (106.7, 112.3) | Ref. |  | 43.9 (37.6, 50.2) | Ref. |  |
| 2 | 109.4 (106.9, 111.9) | 1.00 (0.97, 1.03) | 0.96 | 42.2 (39.3, 45.0) | 0.96 (0.82, 1.12) | 0.61 |
| 3 | 108.7 (106.6, 110.8) | 0.99 (0.96, 1.02) | 0.65 | 41.7 (39.6, 43.9) | 0.95 (0.82, 1.10) | 0.50 |
| 4 | 111.1 (109.3, 112.8) | 1.01 (0.99, 1.04) | 0.34 | 40.9 (39.1, 42.7) | 0.93 (0.81, 1.08) | 0.33 |
| 5 Most deprived | 127.8 (125.8, 129.8) | 1.17 (1.13, 1.20) | <0.01 | 44.9 (43.0, 46.7) | 1.02 (0.89, 1.18) | 0.77 |
| Latin America & Caribbean |  |  |  |  |  |  |
| 1 Least deprived | 119.3 (110.4, 128.2) | Ref. |  | 51.2 (38.8, 63.6) | Ref. |  |
| 2 | 114.1 (105.5, 122.8) | 0.96 (0.86, 1.06) | 0.41 | 51.1 (34.3, 68.0) | 1.00 (0.66, 1.50) | 0.99 |
| 3 | 116.4 (109.9, 123.0) | 0.98 (0.89, 1.07) | 0.61 | 48.2 (40.6, 55.9) | 0.94 (0.71, 1.26) | 0.68 |
| 4 | 111.2 (106.0, 116.3) | 0.93 (0.85, 1.02) | 0.11 | 49.6 (44.4, 54.8) | 0.97 (0.74, 1.26) | 0.81 |
| 5 Most deprived | 123.8 (118.8, 128.9) | 1.04 (0.95, 1.13) | 0.39 | 51.6 (47.2, 56.0) | 1.01 (0.78, 1.30) | 0.96 |
| Middle East & North Africa |  |  |  |  |  |  |
| 1 Least deprived | 116.4 (107.7, 125.1) | Ref. |  | 40.3 (34.3, 46.3) | Ref. |  |
| 2 | 107.5 (100.6, 114.4) | 0.92 (0.84, 1.02) | 0.11 | 49.2 (39.7, 58.8) | 1.22 (0.96, 1.56) | 0.10 |
| 3 | 108.9 (103.2, 114.6) | 0.94 (0.85, 1.02) | 0.15 | 48.9 (43.7, 54.1) | 1.21 (1.01, 1.45) | 0.04 |
| 4 | 124.5 (119.5, 129.5) | 1.07 (0.98, 1.16) | 0.12 | 60.0 (45.9, 74.1) | 1.49 (1.13, 1.96) | <0.01 |
| 5 Most deprived | 150.9 (146.5, 155.3) | 1.30 (1.20, 1.40) | <0.01 | 63.9 (56.9, 70.8) | 1.59 (1.32, 1.90) | <0.01 |
| North America |  |  |  |  |  |  |
| 1 Least deprived | 109.9 (102.5, 117.4) | Ref. |  | 46.3 (36.4, 56.1) | Ref. |  |
| 2 | 89.8 (83.8, 95.8) | 0.82 (0.74, 0.90) | <0.01 | 32.3 (27.9, 36.6) | 0.70 (0.54, 0.90) | <0.01 |
| 3 | 100.9 (92.6, 109.2) | 0.92 (0.83, 1.02) | 0.11 | 37.2 (31.0, 43.5) | 0.80 (0.61, 1.05) | 0.11 |
| 4 | 98.4 (91.0, 105.9) | 0.90 (0.81, 0.99) | 0.03 | 39.1 (32.9, 45.3) | 0.84 (0.65, 1.10) | 0.21 |
| 5 Most deprived | 128.2 (114.5, 141.8) | 1.17 (1.03, 1.32) | 0.02 | 56.6 (37.2, 76.0) | 1.22 (0.82, 1.83) | 0.33 |
| South Asia |  |  |  |  |  |  |
| 1 Least deprived | 135.7 (130.7, 140.7) | Ref. |  | 46.6 (41.7, 51.4) | Ref. |  |
| 2 | 139.1 (134.6, 143.5) | 1.02 (0.98, 1.08) | 0.32 | 47.4 (43.5, 51.3) | 1.02 (0.89, 1.16) | 0.79 |
| 3 | 140.1 (136.6, 143.6) | 1.03 (0.99, 1.08) | 0.16 | 51.9 (48.7, 55.1) | 1.11 (0.99, 1.25) | 0.07 |
| 4 | 145.7 (143.0, 148.4) | 1.07 (1.03, 1.12) | <0.01 | 52.7 (49.4, 56.1) | 1.13 (1.00, 1.28) | 0.04 |
| 5 Most deprived | 180.7 (178.2, 183.2) | 1.33 (1.28, 1.38) | <0.01 | 64.4 (60.5, 68.4) | 1.38 (1.23, 1.56) | <0.01 |
| Sub-Saharan Africa |  |  |  |  |  |  |
| 1 Least deprived | 115.6 (111.2, 120.0) | Ref. |  | 43.8 (39.0, 48.5) | Ref. |  |
| 2 | 118.2 (113.9, 122.6) | 1.02 (0.97, 1.08) | 0.40 | 46.1 (42.0, 50.3) | 1.05 (0.92, 1.21) | 0.46 |
| 3 | 117.1 (113.5, 120.8) | 1.01 (0.97, 1.06) | 0.59 | 44.8 (41.6, 48.1) | 1.02 (0.90, 1.16) | 0.72 |
| 4 | 114.0 (111.5, 116.5) | 0.99 (0.94, 1.03) | 0.53 | 49.9 (46.6, 53.2) | 1.14 (1.01, 1.29) | 0.04 |
| 5 Most deprived | 127.2 (125.1, 129.4) | 1.10 (1.06, 1.15) | <0.01 | 52.1 (49.9, 54.3) | 1.19 (1.06, 1.33) | <0.01 |
| UK |  |  |  |  |  |  |
| 1 Least deprived | 147.3 (146.1, 148.5) | Ref. |  | 46.4 (45.2, 47.7) | Ref. |  |
| 2 | 159.5 (158.3, 160.8) | 1.08 (1.07, 1.09) | <0.01 | 47.6 (46.3, 48.9) | 1.02 (1.00, 1.05) | 0.09 |
| 3 | 168.0 (166.8, 169.3) | 1.14 (1.13, 1.15) | <0.01 | 48.6 (47.3, 49.9) | 1.05 (1.02, 1.08) | <0.01 |
| 4 | 183.4 (182.1, 184.7) | 1.25 (1.23, 1.26) | <0.01 | 50.6 (49.4, 51.8) | 1.09 (1.06, 1.12) | <0.01 |
| 5 Most deprived | 210.8 (209.3, 212.2) | 1.43 (1.42, 1.44) | <0.01 | 54.6 (53.3, 55.8) | 1.18 (1.14, 1.21) | <0.01 |

CI = Confidence interval, IMD = index of multiple deprivation, IRR = incidence rate ratio; *results derived from negative binomial regression models adjusted for year of birth, maternal region of birth, IMD group and maternal region of birth*IMD group interaction terms (regression model results available on request); marginal incidence rates derived from models with year of birth set to mid-study (2011); IRR of admission rates for IMD groups in comparison to the least deprived IMD group, within maternal region groups; ***N* = 4,174,596, AIC = 7796925.19 (compared with AIC = 7797823.04 for model without interaction term); ****N* = 4,174,596, AIC = 3291383.78 (compared with AIC = 3291546.74 for model without interaction term)

Table S11. Estimated incidence rates and IRRs of emergency and planned hospital admissions, by maternal country of birth and IMD group (derived from negative binomial regression models)*

|  | Emergency admissions** | | | Planned admissions*** | | |
| --- | --- | --- | --- | --- | --- | --- |
|  | Incidence rate (95% CI)  per 1000 child-years | IRR (95% CI) | p-value | Incidence rate (95% CI)  per 1000 child-years | IRR (95% CI) | p-value |
| Bangladesh |  |  |  |  |  |  |
| 1 Least deprived | 135.6 (119.3, 151.9) | Ref. |  | 43.1 (29.2, 57.0) | Ref. |  |
| 2 | 143.4 (128.0, 158.8) | 1.06 (0.90, 1.24) | 0.49 | 53.4 (41.1, 65.6) | 1.24 (0.83, 1.84) | 0.29 |
| 3 | 153.9 (142.9, 164.9) | 1.14 (0.99, 1.30) | 0.07 | 55.6 (47.2, 64.1) | 1.29 (0.90, 1.84) | 0.16 |
| 4 | 146.0 (137.6, 154.4) | 1.08 (0.94, 1.23) | 0.28 | 50.0 (44.9, 55.0) | 1.16 (0.83, 1.62) | 0.39 |
| 5 Most deprived | 158.2 (154.1, 162.3) | 1.17 (1.03, 1.32) | 0.01 | 60.4 (55.9, 64.9) | 1.40 (1.01, 1.95) | 0.05 |
| India |  |  |  |  |  |  |
| 1 Least deprived | 132.4 (125.9, 138.9) | Ref. |  | 40.9 (36.2, 45.7) | Ref. |  |
| 2 | 130.5 (124.5, 136.5) | 0.99 (0.92, 1.05) | 0.67 | 39.0 (34.3, 43.6) | 0.95 (0.81, 1.12) | 0.56 |
| 3 | 124.8 (120.5, 129.1) | 0.94 (0.89, 1.00) | 0.05 | 41.3 (37.5, 45.0) | 1.01 (0.87, 1.17) | 0.92 |
| 4 | 121.9 (118.4, 125.5) | 0.92 (0.87, 0.97) | <0.01 | 41.3 (37.9, 44.8) | 1.01 (0.88, 1.16) | 0.90 |
| 5 Most deprived | 145.5 (141.0, 150.1) | 1.10 (1.04, 1.16) | <0.01 | 42.3 (38.5, 46.2) | 1.03 (0.89, 1.20) | 0.66 |
| Nigeria |  |  |  |  |  |  |
| 1 Least deprived | 114.2 (100.0, 128.3) | Ref. |  | 42.0 (33.6, 50.4) | Ref. |  |
| 2 | 111.3 (101.4, 121.3) | 0.98 (0.84, 1.14) | 0.75 | 44.2 (35.4, 52.9) | 1.05 (0.80, 1.39) | 0.73 |
| 3 | 106.8 (98.7, 114.9) | 0.94 (0.81, 1.08) | 0.37 | 41.8 (34.8, 48.8) | 1.00 (0.77, 1.29) | 0.97 |
| 4 | 107.6 (102.9, 112.3) | 0.94 (0.83, 1.07) | 0.38 | 53.3 (47.9, 58.6) | 1.27 (1.02, 1.58) | 0.04 |
| 5 Most deprived | 114.5 (110.5, 118.6) | 1.00 (0.88, 1.14) | 0.96 | 48.7 (43.6, 53.8) | 1.16 (0.93, 1.45) | 0.19 |
| Pakistan |  |  |  |  |  |  |
| 1 Least deprived | 154.7 (142.7, 166.7) | Ref. |  | 61.3 (50.0, 72.6) | Ref. |  |
| 2 | 161.3 (151.6, 171.1) | 1.04 (0.95, 1.15) | 0.40 | 58.9 (49.0, 68.8) | 0.96 (0.75, 1.23) | 0.75 |
| 3 | 164.6 (156.6, 172.7) | 1.06 (0.97, 1.17) | 0.18 | 65.8 (57.9, 73.7) | 1.07 (0.86, 1.34) | 0.52 |
| 4 | 176.5 (171.2, 181.9) | 1.14 (1.05, 1.24) | <0.01 | 61.5 (53.8, 69.2) | 1.00 (0.80, 1.25) | 0.97 |
| 5 Most deprived | 209.8 (205.7, 213.9) | 1.36 (1.25, 1.47) | <0.01 | 75.1 (67.8, 82.3) | 1.22 (1.00, 1.51) | 0.06 |
| Poland |  |  |  |  |  |  |
| 1 Least deprived | 98.3 (93.1, 103.4) | Ref. |  | 38.9 (34.6, 43.2) | Ref. |  |
| 2 | 100.0 (95.7, 104.3) | 1.02 (0.95, 1.09) | 0.60 | 40.3 (35.2, 45.4) | 1.04 (0.88, 1.22) | 0.68 |
| 3 | 99.9 (96.5, 103.4) | 1.02 (0.96, 1.08) | 0.59 | 40.1 (36.4, 43.8) | 1.03 (0.89, 1.19) | 0.68 |
| 4 | 102.6 (99.9, 105.2) | 1.04 (0.99, 1.11) | 0.15 | 39.6 (36.3, 42.9) | 1.02 (0.89, 1.17) | 0.80 |
| 5 Most deprived | 116.4 (113.4, 119.4) | 1.18 (1.12, 1.25) | <0.01 | 40.1 (37.5, 42.7) | 1.03 (0.91, 1.17) | 0.63 |
| UK |  |  |  |  |  |  |
| 1 Least deprived | 147.6 (146.4, 148.8) | Ref. |  | 46.4 (45.1, 47.6) | Ref. |  |
| 2 | 159.8 (158.5, 161.1) | 1.08 (1.07, 1.09) | <0.01 | 47.6 (46.2, 48.9) | 1.03 (1.00, 1.06) | 0.09 |
| 3 | 168.3 (167.0, 169.6) | 1.14 (1.13, 1.15) | <0.01 | 48.6 (47.3, 49.9) | 1.05 (1.02, 1.08) | <0.01 |
| 4 | 183.7 (182.3, 185.1) | 1.24 (1.23, 1.26) | <0.01 | 50.5 (49.3, 51.8) | 1.09 (1.06, 1.12) | <0.01 |
| 5 Most deprived | 211.1 (209.6, 212.7) | 1.43 (1.42, 1.44) | <0.01 | 54.5 (53.2, 55.8) | 1.18 (1.14, 1.21) | <0.01 |

CI = Confidence interval, IMD = index of multiple deprivation, IRR = incidence rate ratio; *results derived from negative binomial regression models adjusted for year of birth, maternal country of birth, IMD group and maternal country of birth*IMD group interaction terms (regression model results available on request); marginal incidence rates derived from models with year of birth set to mid-study (2011); IRR of admission rates for IMD groups in comparison to the least deprived IMD group, within maternal country groups; ***N* = 3,492,139, AIC = 6729769.86 (compared with AIC = 6730123.41 for model without interaction term); ****N* = 3,492,139, AIC = 2775796.38 (compared with AIC = 2775829.28 for model without interaction term)

Table S12. Estimated incidence rates and IRRs of emergency and planned hospital admissions, by parental migration status and IMD group (derived from negative binomial regression models*)

|  | Emergency admissions** | | | Planned admissions*** | | |
| --- | --- | --- | --- | --- | --- | --- |
|  | Incidence rate (95% CI)  per 1000 child-years | IRR (95% CI) | *p*-value | Incidence rate (95% CI)  per 1000 child-years | IRR (95% CI) | *p-*value |
| Mother UK-born & SP UK-born |  |  |  |  |  |  |
| 1 Least deprived | 146.9 (145.7, 148.1) | Ref. |  | 46.4 (45.1, 47.7) | Ref. |  |
| 2 | 158.5 (157.3, 159.8) | 1.08 (1.07, 1.09) | <0.01 | 47.5 (46.2, 48.9) | 1.03 (0.99, 1.06) | 0.12 |
| 3 | 166.4 (165.1, 167.7) | 1.13 (1.12, 1.14) | <0.01 | 48.2 (46.8, 49.5) | 1.04 (1.01, 1.07) | 0.01 |
| 4 | 182.0 (180.7, 183.4) | 1.24 (1.23, 1.25) | <0.01 | 49.7 (48.4, 51.0) | 1.07 (1.04, 1.10) | <0.01 |
| 5 Most deprived | 208.3 (206.8, 209.8) | 1.42 (1.41, 1.43) | <0.01 | 53.0 (51.7, 54.4) | 1.14 (1.11, 1.18) | <0.01 |
| Mother UK-born & SP non-UK-born |  |  |  |  |  |  |
| 1 Least deprived | 130.4 (126.9, 134.0) | Ref. |  | 45.6 (42.3, 48.9) | Ref. |  |
| 2 | 140.7 (137.0, 144.4) | 1.08 (1.04, 1.12) | <0.01 | 44.7 (42.0, 47.5) | 0.98 (0.89, 1.08) | 0.68 |
| 3 | 150.1 (146.5, 153.7) | 1.15 (1.11, 1.19) | <0.01 | 50.1 (46.8, 53.3) | 1.10 (1.00, 1.21) | 0.05 |
| 4 | 163.0 (159.3, 166.7) | 1.25 (1.21, 1.29) | <0.01 | 52.6 (49.6, 55.6) | 1.15 (1.06, 1.26) | <0.01 |
| 5 Most deprived | 199.5 (195.9, 203.0) | 1.53 (1.48, 1.58) | <0.01 | 62.1 (58.9, 65.3) | 1.36 (1.25, 1.48) | <0.01 |
| Mother UK-born (no SP registered) |  |  |  |  |  |  |
| 1 Least deprived | 209.6 (201.8, 217.5) | Ref. |  | 51.1 (45.8, 56.4) | Ref. |  |
| 2 | 221.5 (214.9, 228.1) | 1.06 (1.01, 1.11) | 0.02 | 54.2 (49.8, 58.7) | 1.06 (0.93, 1.21) | 0.37 |
| 3 | 223.0 (217.3, 228.6) | 1.06 (1.02, 1.11) | 0.01 | 55.0 (50.9, 59.2) | 1.08 (0.95, 1.22) | 0.25 |
| 4 | 223.7 (219.4, 228.0) | 1.07 (1.02, 1.11) | <0.01 | 58.4 (55.3, 61.5) | 1.14 (1.02, 1.28) | 0.02 |
| 5 Most deprived | 239.4 (235.9, 242.8) | 1.14 (1.10, 1.19) | <0.01 | 58.3 (55.1, 61.4) | 1.14 (1.02, 1.28) | 0.02 |
| Mother non-UK-born & SP non-UK-born |  |  |  |  |  |  |
| 1 Least deprived | 109.9 (107.5, 112.3) | Ref. |  | 41.1 (38.7, 43.4) | Ref. |  |
| 2 | 108.9 (106.9, 111.0) | 0.99 (0.96, 1.02) | 0.54 | 42.6 (40.2, 45.0) | 1.04 (0.96, 1.12) | 0.35 |
| 3 | 113.0 (111.3, 114.7) | 1.03 (1.00, 1.06) | 0.03 | 44.4 (42.7, 46.1) | 1.08 (1.01, 1.15) | 0.02 |
| 4 | 118.2 (116.7, 119.6) | 1.08 (1.05, 1.10) | <0.01 | 47.4 (45.5, 49.3) | 1.15 (1.08, 1.23) | <0.01 |
| 5 Most deprived | 141.3 (139.9, 142.7) | 1.29 (1.26, 1.32) | <0.01 | 54.0 (52.1, 56.0) | 1.32 (1.23, 1.40) | <0.01 |
| Mother non-UK-born & SP UK-born |  |  |  |  |  |  |
| 1 Least deprived | 120.8 (118.0, 123.6) | Ref. |  | 49.1 (42.7, 55.5) | Ref. |  |
| 2 | 124.4 (121.5, 127.3) | 1.03 (1.00, 1.06) | 0.07 | 44.6 (41.6, 47.5) | 0.91 (0.79, 1.04) | 0.18 |
| 3 | 128.8 (125.9, 131.8) | 1.07 (1.03, 1.10) | <0.01 | 47.2 (43.6, 50.7) | 0.96 (0.83, 1.11) | 0.59 |
| 4 | 138.6 (135.8, 141.5) | 1.15 (1.11, 1.18) | <0.01 | 47.7 (43.6, 51.8) | 0.97 (0.84, 1.13) | 0.72 |
| 5 Most deprived | 174.5 (171.0, 177.9) | 1.44 (1.40, 1.49) | <0.01 | 59.4 (55.3, 63.4) | 1.21 (1.05, 1.39) | 0.01 |
| Mother non-UK-born (sole registration) |  |  |  |  |  |  |
| 1 Least deprived | 120.0 (105.4, 134.7) | Ref. |  | 28.6 (20.9, 36.4) | Ref. |  |
| 2 | 132.5 (120.9, 144.1) | 1.10 (0.95, 1.28) | 0.20 | 44.2 (35.1, 53.3) | 1.54 (1.10, 2.16) | 0.01 |
| 3 | 134.6 (126.3, 143.0) | 1.12 (0.98, 1.29) | 0.10 | 46.9 (40.1, 53.6) | 1.64 (1.21, 2.22) | <0.01 |
| 4 | 124.4 (119.3, 129.5) | 1.04 (0.91, 1.18) | 0.59 | 51.9 (46.3, 57.4) | 1.81 (1.36, 2.42) | <0.01 |
| 5 Most deprived | 141.2 (136.7, 145.8) | 1.18 (1.04, 1.33) | 0.01 | 50.2 (46.4, 54.0) | 1.75 (1.33, 2.32) | <0.01 |

CI = Confidence interval, IMD = index of multiple deprivation, IRR = incidence rate ratio, SP = second parent *results derived from negative binomial regression models adjusted for year of birth, maternal region of birth, IMD group and maternal region of birth*IMD group interaction terms (regression model results available on request); marginal incidence rates derived from models with year of birth set to mid-study (2011); IRR of admission rates for IMD groups in comparison to the least deprived IMD group, within maternal region groups; ***N* = 4,174,596, AIC = 7796685.71 (compared with AIC = 7797359.99 for model without interaction term); ****N* = 4,174,596, AIC = 3291959.24 (compared with AIC = 3292159.78 for model without interaction term)
