# Supplementary material for: Parental migration, socioeconomic deprivation and hospital admissions in preschool children in England: national birth cohort study, 2008 to 2014
Source: BMC Med. 2024 Sep 27;22:416. doi: 10.1186/s12916-024-03619-1 (PMC11438240; doi:10.1186/s12916-024-03619-1)
Supplement: Supplementary file 7 — Additional file 7. Secondary outcome results (Tables S13-S16). Table S13 – Observed rates. Table S14—Estimated rates by maternal region of birth. Table S15 – Estimated rates by maternal country of birth. Table S16 – Estimated rates by parental migration status. [file 12916_2024_3619_MOESM7_ESM.docx]

## Additional File 7: Secondary results – Admissions for acute infections, neonatal feeding difficulties and tooth extractions for caries

**Table S13. Observed numbers and rates (per 1000 child-years) of admissions with specified diagnoses, by maternal region of birth, maternal country of birth, migration status of parents and IMD group: England, births from 2008 to 2014**

|  | **Acute infections** | | **Neonatal feeding difficulties** | | **Tooth extraction for caries** | |
| --- | --- | --- | --- | --- | --- | --- |
|  | **N (% of cases)** | **Rate per 1000 child-years (95% CI)** | **N (% of cases)** | **Rate per 1000 child-years (95% CI)** | **N (% of cases)** | **Rate per 1000 child-years (95% CI)** |
| **Overall** | 795867 (100.0) | 60.1 (60.0,60.2) | 119742 (100.0) | 60.5 (60.1,60.8) | 18999 (100.0) | 3.10 (3.06,3.14) |
| **Maternal region of birth** |  |  |  |  |  |  |
| East-Asia & Pacific | 10756 (1.4) | 35.5 (34.8,36.1) | 4440 (3.7) | 99.3 (96.4,102.3) | 621 (3.3) | 4.39 (4.06,4.75) |
| Europe (excl. UK) & Central Asia | 44492 (5.6) | 39.1 (38.7,39.5) | 13617 (11.4) | 75.9 (74.7,77.2) | 2244 (11.8) | 4.42 (4.24,4.61) |
| Latin America & Caribbean | 4688 (0.6) | 36.3 (35.3,37.4) | 1011 (0.8) | 52.8 (49.6,56.2) | 224 (1.2) | 3.72 (3.26,4.24) |
| Middle East & North Africa | 8440 (1.1) | 44.5 (43.6,45.5) | 1947 (1.6) | 67.8 (64.8,70.9) | 515 (2.7) | 5.91 (5.42,6.44) |
| North America | 2321 (0.3) | 32.8 (31.4,34.1) | 741 (0.6) | 69.5 (64.7,74.7) | 40 (0.2) | 1.22 (0.90,1.67) |
| South Asia | 51571 (6.5) | 55.1 (54.6,55.6) | 10035 (8.4) | 71.4 (70.0,72.8) | 2292 (12.1) | 5.31 (5.10,5.54) |
| Sub-Saharan Africa | 27746 (3.5) | 41.6 (41.2,42.1) | 3404 (2.8) | 34.9 (33.8,36.1) | 971 (5.1) | 3.10 (2.91,3.30) |
| UK | 645853 (81.2) | 65.8 (65.7,66.0) | 84547 (70.6) | 57.9 (57.5,58.3) | 12092 (63.6) | 2.65 (2.61,2.70) |
| **Maternal country of birth** |  |  |  |  |  |  |
| Bangladesh | 8623 (1.1) | 52.7 (51.6,53.9) | 1531 (1.3) | 63.5 (60.4,66.8) | 511 (2.7) | 6.68 (6.13,7.29) |
| India | 11596 (1.5) | 43.4 (42.6,44.2) | 3357 (2.8) | 82.6 (79.8,85.4) | 351 (1.8) | 2.89 (2.60,3.21) |
| Nigeria | 4967 (0.6) | 36.8 (35.7,37.8) | 603 (0.5) | 29.7 (27.4,32.1) | 59 (0.3) | 0.95 (0.74,1.23) |
| Pakistan | 25481 (3.2) | 69.5 (68.7,70.4) | 3500 (2.9) | 63.8 (61.8,66.0) | 999 (5.3) | 5.89 (5.54,6.27) |
| Poland | 13308 (1.7) | 35.6 (35.0,36.2) | 4759 (4.0) | 81.9 (79.6,84.3) | 797 (4.2) | 4.74 (4.42,5.08) |
| **Migration status of parents** |  |  |  |  |  |  |
| Both UK-born | 550274 (69.1) | 65.2 (65.1,65.4) | 72899 (60.9) | 58.0 (57.6,58.4) | 9338 (49.1) | 2.39 (2.34,2.44) |
| Mother UK-born & SP non-UK-born | 44247 (5.6) | 58.2 (57.6,58.7) | 7061 (5.9) | 62.7 (61.3,64.2) | 1190 (6.3) | 3.35 (3.17,3.55) |
| Mother UK-born (sole registration) | 51332 (6.4) | 83.4 (82.6,84.1) | 4587 (3.8) | 51.3 (49.8,52.8) | 1564 (8.2) | 5.38 (5.12,5.66) |
| Both non-UK-born | 102895 (12.9) | 42.2 (41.9,42.4) | 25871 (21.6) | 69.4 (68.6,70.3) | 5374 (28.3) | 4.83 (4.70,4.96) |
| Mother non-UK-born & SP UK-born | 40440 (5.1) | 47.6 (47.1,48.1) | 8331 (7.0) | 65.8 (64.4,67.2) | 1124 (5.9) | 2.85 (2.69,3.02) |
| Mother non-UK-born (sole registration) | 6679 (0.8) | 46.4 (45.3,47.5) | 993 (0.8) | 46.2 (43.4,49.2) | 409 (2.2) | 6.15 (5.58,6.78) |
| **IMD groups** |  |  |  |  |  |  |
| 1 Least deprived | 102246 (12.8) | 50.7 (50.3,51.0) | 18501 (15.5) | 61.8 (60.9,62.7) | 1009 (5.3) | 1.07 (1.01,1.14) |
| 2 | 118259 (14.9) | 55.4 (55.1,55.8) | 20243 (16.9) | 63.1 (62.3,64.0) | 1658 (8.7) | 1.68 (1.60,1.77) |
| 3 | 138383 (17.4) | 56.4 (56.1,56.7) | 22575 (18.9) | 61.3 (60.5,62.1) | 2625 (13.8) | 2.32 (2.23,2.41) |
| 4 | 179270 (22.5) | 60.0 (59.8,60.3) | 26793 (22.4) | 59.8 (59.1,60.6) | 4844 (25.5) | 3.51 (3.42,3.61) |
| 5 Most deprived | 257709 (32.4) | 70.5 (70.3,70.8) | 31630 (26.4) | 58.1 (57.5,58.8) | 8863 (46.6) | 5.23 (5.13,5.34) |

CI = Confidence interval, IMD = index of multiple deprivation, SP=second parent

Table S14. Estimated incidence rates (per 1000 child-years) and IRRs of hospital admissions with specified diagnoses, by maternal region of birth and IMD group (derived from negative binomial/Poisson regression models)*

|  | Acute infection** | | | Neonatal feeding difficulties*** | | | Tooth extraction for caries^a^ | | |
| --- | --- | --- | --- | --- | --- | --- | --- | --- | --- |
|  | Incidence rate  (95% CI) | IRR (95% CI) | p-value | Incidence rate (95% CI) | IRR (95% CI) | p-value | Incidence rate (95% CI) | IRR (95% CI) | p-value |
| East-Asia & Pacific |  |  |  |  |  |  |  |  |  |
| 1 Least deprived | 32.8 (30.9,34.8) | Ref. |  | 91.3 (83.6,98.9) | Ref. |  | 1.13 (0.78, 1.48) | Ref. |  |
| 2 | 33.2 (31.2,35.1) | 1.01 (0.93,1.10) | 0.82 | 94.4 (86.5,102.3) | 1.03 (0.92,1.16) | 0.57 | 1.80 (1.36, 2.23) | 1.59 (1.08,2.34) | 0.02 |
| 3 | 34.8 (32.8,36.7) | 1.06 (0.97,1.15) | 0.18 | 92.8 (85.9,99.7) | 1.02 (0.91,1.14) | 0.76 | 1.73 (1.35, 2.12) | 1.53 (1.05,2.23) | 0.03 |
| 4 | 36.7 (35.0,38.5) | 1.12 (1.04,1.21) | <0.01 | 96.5 (90.1,103.0) | 1.06 (0.95,1.17) | 0.30 | 3.28 (2.77, 3.79) | 2.90 (2.07,4.07) | <0.01 |
| 5 Most deprived | 44.6 (42.6,46.6) | 1.36 (1.26,1.46) | <0.01 | 101.6 (94.5,108.7) | 1.11 (1.00,1.24) | 0.05 | 4.13 (3.55, 4.72) | 3.66 (2.62,5.10) | <0.01 |
| Europe (excl. UK) & Central Asia |  |  |  |  |  |  |  |  |  |
| 1 Least deprived | 37.1 (35.7,38.4) | Ref. |  | 68.9 (64.9,72.9) | Ref. |  | 0.98 (0.78, 1.18) | Ref. |  |
| 2 | 37.2 (36.0,38.5) | 1.00 (0.96,1.05) | 0.85 | 65.8 (62.3,69.2) | 0.95 (0.88,1.03) | 0.22 | 1.56 (1.32, 1.81) | 1.60 (1.24,2.05) | <0.01 |
| 3 | 36.8 (35.8,37.9) | 0.99 (0.95,1.04) | 0.79 | 68.8 (65.7,71.9) | 1.00 (0.93,1.07) | 0.97 | 2.19 (1.94, 2.44) | 2.23 (1.78,2.81) | <0.01 |
| 4 | 37.5 (36.6,38.4) | 1.01 (0.97,1.06) | 0.62 | 72.4 (69.6,75.2) | 1.05 (0.98,1.12) | 0.14 | 2.87 (2.61, 3.13) | 2.93 (2.36,3.64) | <0.01 |
| 5 Most deprived | 45.8 (44.8,46.9) | 1.24 (1.19,1.29) | <0.01 | 76.2 (73.5,79.0) | 1.11 (1.04,1.18) | <0.01 | 3.99 (3.67, 4.31) | 4.08 (3.30,5.04) | <0.01 |
| Latin America & Caribbean |  |  |  |  |  |  |  |  |  |
| 1 Least deprived | 39.7 (35.2,44.2) | Ref. |  | 57.7 (46.6,68.9) | Ref. |  | 0.73 (0.19, 1.27) | Ref. |  |
| 2 | 38.6 (34.2,43.1) | 0.97 (0.83,1.14) | 0.74 | 59.4 (48.4,70.4) | 1.03 (0.79,1.34) | 0.84 | 0.78 (0.18, 1.38) | 1.07 (0.37,3.11) | 0.91 |
| 3 | 36.3 (33.2,39.4) | 0.91 (0.79,1.05) | 0.21 | 59.9 (50.7,69.1) | 1.04 (0.81,1.33) | 0.77 | 1.70 (1.01, 2.39) | 2.33 (1.01,5.41) | 0.05 |
| 4 | 34.3 (31.9,36.6) | 0.86 (0.76,0.99) | 0.03 | 45.6 (39.6,51.7) | 0.79 (0.63,1.00) | 0.05 | 2.71 (2.10, 3.32) | 3.72 (1.72,8.05) | <0.01 |
| 5 Most deprived | 40.8 (38.4,43.1) | 1.03 (0.90,1.17) | 0.68 | 45.6 (40.2,51.0) | 0.79 (0.63,0.99) | 0.04 | 2.76 (2.17, 3.34) | 3.79 (1.76,8.16) | <0.01 |
| Middle East & North Africa |  |  |  |  |  |  |  |  |  |
| 1 Least deprived | 37.9 (33.7,42.2) | Ref. |  | 78.3 (65.1,91.6) | Ref. |  | 0.79 (0.18, 1.40) | Ref. |  |
| 2 | 34.4 (31.1,37.7) | 0.91 (0.78,1.05) | 0.19 | 62.5 (52.7,72.3) | 0.80 (0.63,1.00) | 0.05 | 2.45 (1.62, 3.28) | 3.11 (1.34,7.23) | 0.01 |
| 3 | 36.5 (33.8,39.2) | 0.96 (0.84,1.10) | 0.57 | 58.5 (50.9,66.2) | 0.75 (0.60,0.92) | 0.01 | 3.25 (2.47, 4.02) | 4.12 (1.84,9.25) | <0.01 |
| 4 | 43.0 (40.6,45.4) | 1.13 (1.00,1.28) | 0.05 | 61.8 (55.6,68.1) | 0.79 (0.65,0.96) | 0.02 | 3.86 (3.19, 4.52) | 4.89 (2.22,10.79) | <0.01 |
| 5 Most deprived | 54.9 (52.7,57.2) | 1.45 (1.29,1.63) | <0.01 | 66.6 (61.6,71.6) | 0.85 (0.71,1.02) | 0.08 | 4.02 (3.49, 4.55) | 5.10 (2.33,11.16) | <0.01 |
| North America |  |  |  |  |  |  |  |  |  |
| 1 Least deprived | 37.0 (33.2,40.8) | Ref. |  | 69.6 (58.9,80.3) | Ref. |  | 0.46 (0.09, 0.82) | Ref. |  |
| 2 | 28.3 (25.2,31.5) | 0.77 (0.66,0.89) | <0.01 | 66.4 (55.2,77.7) | 0.95 (0.76,1.20) | 0.69 | 0.46 (0.09, 0.83) | 1.00 (0.32,3.11) | 0.99 |
| 3 | 32.3 (28.4,36.2) | 0.87 (0.75,1.02) | 0.09 | 65.4 (54.4,76.5) | 0.94 (0.75,1.18) | 0.59 | 0.59 (0.15, 1.02) | 1.28 (0.43,3.80) | 0.66 |
| 4 | 32.0 (28.2,35.8) | 0.86 (0.74,1.01) | 0.07 | 68.8 (57.0,80.6) | 0.99 (0.79,1.24) | 0.92 | 1.01 (0.41, 1.61) | 2.21 (0.82,5.98) | 0.12 |
| 5 Most deprived | 45.7 (39.3,52.1) | 1.23 (1.04,1.47) | 0.02 | 58.0 (44.1,71.9) | 0.83 (0.63,1.11) | 0.21 | 1.62 (0.52, 2.72) | 3.55 (1.25,10.10) | 0.02 |
| South Asia |  |  |  |  |  |  |  |  |  |
| 1 Least deprived | 48.0 (45.4,50.6) | Ref. |  | 70.1 (63.7,76.5) | Ref. |  | 1.31 (0.90, 1.72) | Ref. |  |
| 2 | 47.7 (45.5,49.9) | 0.99 (0.92,1.07) | 0.84 | 74.6 (69.1,80.1) | 1.06 (0.95,1.19) | 0.29 | 2.09 (1.68, 2.50) | 1.60 (1.11,2.30) | 0.01 |
| 3 | 47.5 (45.8,49.3) | 0.99 (0.93,1.06) | 0.75 | 69.3 (65.5,73.1) | 0.99 (0.89,1.10) | 0.83 | 2.87 (2.52, 3.23) | 2.20 (1.58,3.06) | <0.01 |
| 4 | 50.7 (49.5,52.0) | 1.06 (1.00,1.12) | 0.07 | 64.5 (61.7,67.2) | 0.92 (0.83,1.02) | 0.10 | 3.18 (2.89, 3.47) | 2.43 (1.77,3.35) | <0.01 |
| 5 Most deprived | 67.2 (66.0,68.5) | 1.40 (1.32,1.48) | <0.01 | 69.2 (66.8,71.6) | 0.99 (0.90,1.09) | 0.80 | 3.60 (3.33, 3.87) | 2.75 (2.01,3.77) | <0.01 |
| Sub-Saharan Africa |  |  |  |  |  |  |  |  |  |
| 1 Least deprived | 41.6 (39.4,43.9) | Ref. |  | 44.4 (39.4,49.4) | Ref. |  | 0.91 (0.62, 1.21) | Ref. |  |
| 2 | 41.0 (38.8,43.2) | 0.98 (0.91,1.06) | 0.69 | 50.6 (45.2,56.0) | 1.14 (0.98,1.33) | 0.09 | 0.72 (0.46, 0.98) | 0.79 (0.49,1.26) | 0.32 |
| 3 | 40.7 (38.9,42.6) | 0.98 (0.91,1.05) | 0.53 | 38.0 (34.4,41.6) | 0.86 (0.74,0.99) | 0.04 | 1.14 (0.88, 1.40) | 1.25 (0.84,1.84) | 0.27 |
| 4 | 40.4 (39.1,41.6) | 0.97 (0.91,1.03) | 0.33 | 28.9 (26.7,31.1) | 0.65 (0.57,0.74) | <0.01 | 2.02 (1.76, 2.28) | 2.21 (1.58,3.11) | <0.01 |
| 5 Most deprived | 47.5 (46.4,48.6) | 1.14 (1.08,1.21) | <0.01 | 30.1 (28.3,31.9) | 0.68 (0.60,0.77) | <0.01 | 2.26 (2.04, 2.49) | 2.47 (1.78,3.44) | <0.01 |
| UK |  |  |  |  |  |  |  |  |  |
| 1 Least deprived | 55.6 (55.0,56.2) | Ref. |  | 57.6 (56.4,58.9) | Ref. |  | 0.57 (0.52, 0.62) | Ref. |  |
| 2 | 61.7 (61.1,62.4) | 1.11 (1.10,1.12) | <0.01 | 58.6 (57.3,59.8) | 1.02 (0.99,1.04) | 0.19 | 0.88 (0.82, 0.95) | 1.56 (1.42,1.71) | <0.01 |
| 3 | 64.5 (63.8,65.1) | 1.16 (1.15,1.17) | <0.01 | 56.3 (55.1,57.6) | 0.98 (0.95,1.00) | 0.06 | 1.15 (1.07, 1.22) | 2.02 (1.85,2.20) | <0.01 |
| 4 | 71.6 (70.9,72.3) | 1.29 (1.27,1.30) | <0.01 | 55.0 (53.8,56.2) | 0.95 (0.93,0.98) | <0.01 | 1.76 (1.66, 1.86) | 3.10 (2.86,3.36) | <0.01 |
| 5 Most deprived | 84.0 (83.3,84.8) | 1.51 (1.49,1.53) | <0.01 | 52.0 (51.0,53.1) | 0.90 (0.88,0.92) | <0.01 | 2.95 (2.80, 3.11) | 5.21 (4.82,5.62) | <0.01 |

CI = Confidence interval, IMD = index of multiple deprivation, IRR = incidence rate ratio; *results derived from negative binomial/Poisson regression models adjusted for year of birth, maternal region of birth, IMD group and maternal region of birth*IMD group interaction term (regression model results available on request); marginal incidence rates derived from models with year of birth set to mid-study (2011); IRR of admission rates for IMD groups in comparison to the least deprived IMD group, within maternal region groups; **Negative binomial regression, *N* = 4,174,596, AIC = 4206765.23 (compared with AIC = 4207230.40 for model without interaction term); *** Negative binomial regression, *N* = 4,174,596, AIC = 1088294.95 (compared with AIC = 1088471.92 for model without interaction term); ^a^Poisson regression, *N* = 2,973,284, AIC = 213907.46 (compared with AIC = 214088.94 for model without interaction term)

Table S15. Estimated incidence rates (per 1000 child-years) and IRRs of hospital admissions with specified diagnoses, by maternal country of birth and IMD group (derived from negative binomial/Poisson regression models)*

|  | Acute infections** | | | Feeding difficulties and jaundice*** | | | Tooth extractions for caries^a^ | | |
| --- | --- | --- | --- | --- | --- | --- | --- | --- | --- |
|  | Incidence rate (95% CI) | IRR (95% CI) | p-value | Incidence rate  (95% CI) | IRR (95% CI) | p-value | Incidence rate  (95% CI) | IRR (95% CI) | p-value |
| Bangladesh | |  |  |  |  |  |  |  |  |
| 1 Least deprived | 46.9 (38.2,55.6) | Ref. |  | 41.7 (25.3,58.2) | Ref. |  | 2.16 (0.57,3.76) | Ref. |  |
| 2 | 50.9 (41.5,60.2) | 1.08 (0.84,1.41) | 0.54 | 65.9 (49.0,82.9) | 1.58 (0.99,2.53) | 0.06 | 2.28 (0.99,3.58) | 1.06 (0.42,2.66) | 0.91 |
| 3 | 52.0 (46.7,57.2) | 1.11 (0.90,1.37) | 0.33 | 57.4 (46.1,68.6) | 1.37 (0.88,2.13) | 0.16 | 3.28 (2.21,4.36) | 1.52 (0.68,3.38) | 0.31 |
| 4 | 52.1 (49.0,55.2) | 1.11 (0.91,1.35) | 0.29 | 51.1 (45.0,57.2) | 1.23 (0.81,1.85) | 0.33 | 3.72 (2.97,4.47) | 1.72 (0.80,3.67) | 0.16 |
| 5 Most deprived | 57.7 (55.5,59.8) | 1.23 (1.02,1.49) | 0.03 | 64.7 (60.3,69.2) | 1.55 (1.04,2.31) | 0.03 | 3.97 (3.49,4.45) | 1.83 (0.87,3.85) | 0.11 |
| India | |  |  |  |  |  |  |  |  |
| 1 Least deprived | 47.7 (44.1,51.2) | Ref. |  | 75.5 (66.1,84.9) | Ref. |  | 0.77 (0.35,1.18) | Ref. |  |
| 2 | 44.5 (41.6,47.3) | 0.93 (0.85,1.03) | 0.17 | 87.4 (78.6,96.2) | 1.16 (0.99,1.36) | 0.07 | 0.88 (0.51,1.24) | 1.14 (0.58,2.26) | 0.70 |
| 3 | 42.0 (39.7,44.2) | 0.88 (0.80,0.96) | 0.01 | 76.4 (70.2,82.6) | 1.01 (0.87,1.17) | 0.87 | 1.53 (1.14,1.92) | 1.99 (1.10,3.60) | 0.02 |
| 4 | 40.5 (38.7,42.3) | 0.85 (0.78,0.93) | 0.00 | 72.2 (67.5,77.0) | 0.96 (0.83,1.10) | 0.53 | 1.93 (1.58,2.27) | 2.51 (1.43,4.42) | <0.01 |
| 5 Most deprived | 50.4 (48.2,52.6) | 1.06 (0.97,1.15) | 0.20 | 83.7 (77.7,89.6) | 1.11 (0.96,1.28) | 0.15 | 2.06 (1.67,2.46) | 2.69 (1.53,4.76) | <0.01 |
| Nigeria | |  |  |  |  |  |  |  |  |
| 1 Least deprived | 42.7 (35.6,49.9) | Ref. |  | 27.9 (16.8,39.0) | Ref. |  | 1.00 (0.02,1.97) | Ref. |  |
| 2 | 36.4 (31.0,41.7) | 0.85 (0.68,1.06) | 0.16 | 27.6 (17.4,37.8) | 0.99 (0.58,1.68) | 0.97 | 0.18 (-0.17,0.53) | 0.18 (0.02,1.62) | 0.13 |
| 3 | 34.9 (31.4,38.5) | 0.82 (0.67,0.99) | 0.04 | 30.3 (23.1,37.5) | 1.09 (0.68,1.72) | 0.72 | 0.57 (0.15,0.99) | 0.57 (0.17,1.93) | 0.37 |
| 4 | 36.4 (34.0,38.7) | 0.85 (0.71,1.02) | 0.08 | 28.6 (24.1,33.2) | 1.03 (0.67,1.57) | 0.91 | 0.49 (0.25,0.73) | 0.49 (0.16,1.46) | 0.20 |
| 5 Most deprived | 40.2 (38.2,42.1) | 0.94 (0.79,1.12) | 0.48 | 27.2 (23.8,30.6) | 0.97 (0.64,1.48) | 0.90 | 0.57 (0.37,0.77) | 0.57 (0.20,1.61) | 0.29 |
| Pakistan | | |  |  |  |  |  |  |  |
| 1 Least deprived | 54.7 (48.8,60.7) | Ref. |  | 62.7 (50.8,74.7) | Ref. |  | 1.41 (0.53,2.29) | Ref. |  |
| 2 | 58.4 (53.4,63.4) | 1.07 (0.93,1.22) | 0.36 | 58.4 (49.2,67.6) | 0.93 (0.73,1.19) | 0.57 | 3.20 (2.25,4.15) | 2.27 (1.14,4.50) | 0.02 |
| 3 | 58.8 (54.6,62.9) | 1.07 (0.94,1.22) | 0.28 | 62.2 (55.8,68.7) | 0.99 (0.80,1.23) | 0.94 | 3.10 (2.48,3.71) | 2.19 (1.15,4.20) | 0.02 |
| 4 | 66.0 (63.4,68.6) | 1.20 (1.07,1.35) | <0.01 | 57.7 (53.4,61.9) | 0.92 (0.75,1.13) | 0.41 | 3.33 (2.87,3.79) | 2.36 (1.25,4.44) | 0.01 |
| 5 Most deprived | 81.0 (78.9,83.0) | 1.48 (1.32,1.65) | <0.01 | 61.9 (58.7,65.0) | 0.99 (0.81,1.20) | 0.89 | 3.48 (3.14,3.82) | 2.46 (1.32,4.60) | <0.01 |
| Poland | |  |  |  |  |  |  |  |  |
| 1 Least deprived | 32.8 (30.2,35.3) | Ref. |  | 77.4 (68.8,86.0) | Ref. |  | 1.49 (0.98,2.01) | Ref. |  |
| 2 | 34.6 (32.4,36.7) | 1.06 (0.96,1.17) | 0.29 | 70.4 (63.5,77.4) | 0.91 (0.79,1.05) | 0.21 | 1.83 (1.36,2.31) | 1.23 (0.80,1.87) | 0.34 |
| 3 | 33.7 (32.1,35.3) | 1.03 (0.94,1.13) | 0.54 | 72.9 (67.5,78.3) | 0.94 (0.83,1.07) | 0.38 | 2.63 (2.19,3.07) | 1.76 (1.21,2.56) | <0.01 |
| 4 | 34.1 (32.8,35.5) | 1.04 (0.96,1.14) | 0.36 | 75.7 (71.2,80.3) | 0.98 (0.86,1.11) | 0.74 | 2.72 (2.34,3.10) | 1.82 (1.26,2.61) | <0.01 |
| 5 Most deprived | 41.7 (40.2,43.2) | 1.27 (1.17,1.39) | <0.01 | 82.5 (77.9,87.1) | 1.07 (0.94,1.20) | 0.30 | 3.39 (2.96,3.81) | 2.27 (1.58,3.24) | <0.01 |
| UK | |  |  |  |  |  |  |  |  |
| 1 Least deprived | 55.7 (55.1,56.4) | Ref. |  | 57.1 (55.8,58.4) | Ref. |  | 0.54 (0.49,0.59) | Ref. |  |
| 2 | 61.8 (61.2,62.5) | 1.00 (1.00,1.00) | <0.01 | 58.0 (56.7,59.3) | 1.02 (0.99,1.04) | 0.19 | 0.84 (0.78,0.91) | 1.56 (1.42,1.71) | <0.01 |
| 3 | 64.6 (63.9,65.3) | 0.00 (0.00,0.00) | <0.01 | 55.8 (54.6,57.1) | 0.98 (0.95,1.00) | 0.06 | 1.09 (1.02,1.17) | 2.02 (1.85,2.20) | <0.01 |
| 4 | 71.8 (71.1,72.5) | 0.00 (0.00,0.00) | <0.01 | 54.5 (53.3,55.7) | 0.95 (0.93,0.98) | <0.01 | 1.68 (1.57,1.79) | 3.10 (2.86,3.36) | <0.01 |
| 5 Most deprived | 84.2 (83.4,85.0) | 0.00 (0.00,0.00) | <0.01 | 51.6 (50.4,52.7) | 0.90 (0.88,0.92) | <0.01 | 2.82 (2.65,2.99) | 5.21 (4.82,5.62) | <0.01 |

CI = Confidence interval, IMD = index of multiple deprivation, IRR = incidence rate ratio; *results derived from negative binomial/Poisson regression models adjusted for year of birth, maternal region of birth, IMD group and maternal region of birth*IMD group interaction term (regression model results available on request); marginal incidence rates derived from models with year of birth set to mid-study (2011); IRR of admission rates for IMD groups in comparison to the least deprived IMD group, within maternal region groups; **Negative binomial regression, *N* = 3,492,139, AIC = 3685534.62 (compared with AIC = 3685728.14 for model without interaction term); ***Negative binomial regression, *N* = 3,492,139, AIC = 899210.58 (compared with AIC = 899254.24 for model without interaction term); ^a^Poisson regression, *N* = 2,496,912, AIC = 168449.75 (compared with AIC = 168621.82 for model without interaction term)

Table S16. Estimated incidence rates (per 1000 child-years) and IRRs of hospital admissions with specified diagnoses, by migration status of parents and IMD group (derived from negative binomial/Poisson regression models)*

|  | Acute infections** | | | Feeding difficulties and jaundice*** | | | Tooth extractions for caries^a^ | | |
| --- | --- | --- | --- | --- | --- | --- | --- | --- | --- |
|  | Incidence rate (95% CI) | IRR (95% CI) | p-value | Incidence rate  (95% CI) | IRR (95% CI) | p-value | Incidence rate  (95% CI) | IRR (95% CI) | p-value |
| Mother UK-born & SP UK-born | |  |  |  |  |  |  |  |  |
| 1 Least deprived | 55.7 (55.1, 56.3) | Ref. |  | 57.4 (56.1, 58.7) | Ref. |  | 0.54 (0.49, 0.59) | Ref. |  |
| 2 | 61.6 (61.0, 62.3) | 1.11 (1.09, 1.12) | <0.01 | 58.5 (57.2, 59.8) | 1.02 (0.99, 1.05) | 0.13 | 0.82 (0.75, 0.88) | 1.52 (1.38, 1.68) |  |
| 3 | 64.2 (63.5, 64.9) | 1.15 (1.14, 1.17) | <0.01 | 56.5 (55.2, 57.7) | 0.98 (0.96, 1.01) | 0.22 | 1.07 (0.99, 1.14) | 1.99 (1.81, 2.18) | <0.01 |
| 4 | 71.8 (71.0, 72.5) | 1.29 (1.27, 1.30) | <0.01 | 55.0 (53.8, 56.2) | 0.96 (0.94, 0.98) | <0.01 | 1.62 (1.52, 1.72) | 3.00 (2.75, 3.28) | <0.01 |
| 5 Most deprived | 83.8 (83.0, 84.6) | 1.50 (1.49, 1.52) | <0.01 | 51.7 (50.5, 52.8) | 0.90 (0.88, 0.92) | <0.01 | 2.81 (2.65, 2.96) | 5.22 (4.81, 5.68) | <0.01 |
| Mother UK-born & SP non-UK-born | |  |  |  |  |  |  |  |  |
| 1 Least deprived | 46.5 (44.7, 48.4) | Ref. |  | 61.3 (57.0, 65.6) | Ref. |  | 0.64 (0.46, 0.82) | Ref. |  |
| 2 | 50.5 (48.6, 52.3) | 1.08 (1.03, 1.14) | <0.01 | 61.8 (57.6, 66.0) | 1.01 (0.92, 1.11) | 0.88 | 1.13 (0.89, 1.37) | 1.77 (1.25, 2.51) | <0.01 |
| 3 | 52.8 (51.0, 54.5) | 1.13 (1.08, 1.19) | <0.01 | 59.1 (55.3, 62.8) | 0.96 (0.88, 1.06) | 0.42 | 1.25 (1.02, 1.48) | 1.96 (1.41, 2.74) | <0.01 |
| 4 | 58.7 (56.9, 60.4) | 1.26 (1.20, 1.32) | <0.01 | 58.6 (55.4, 61.8) | 0.96 (0.88, 1.04) | 0.30 | 2.09 (1.82, 2.36) | 3.28 (2.42, 4.44) | <0.01 |
| 5 Most deprived | 76.5 (74.7, 78.2) | 1.64 (1.57, 1.72) | <0.01 | 60.9 (58.1, 63.7) | 0.99 (0.92, 1.08) | 0.88 | 3.15 (2.86, 3.45) | 4.95 (3.70, 6.62) | <0.01 |
| Mother UK-born (sole registration) | |  |  |  |  |  |  |  |  |
| 1 Least deprived | 78.3 (74.4, 82.2) | Ref. |  | 59.1 (52.3, 65.9) | Ref. |  | 1.68 (1.21, 2.14) | Ref. |  |
| 2 | 85.0 (81.6, 88.4) | 1.09 (1.02, 1.16) | 0.01 | 54.8 (49.6, 60.0) | 0.93 (0.80, 1.07) | 0.31 | 2.35 (1.90, 2.80) | 1.40 (1.01, 1.95) | 0.04 |
| 3 | 85.7 (82.9, 88.5) | 1.09 (1.03, 1.16) | <0.01 | 50.0 (46.0, 54.1) | 0.85 (0.74, 0.97) | 0.02 | 2.47 (2.08, 2.85) | 1.47 (1.08, 2.01) | 0.02 |
| 4 | 85.2 (83.1, 87.3) | 1.09 (1.03, 1.15) | <0.01 | 50.8 (47.5, 54.0) | 0.86 (0.75, 0.98) | 0.02 | 3.09 (2.75, 3.44) | 1.84 (1.38, 2.47) | <0.01 |
| 5 Most deprived | 93.1 (91.3, 94.9) | 1.19 (1.13, 1.25) | <0.01 | 46.3 (44.0, 48.6) | 0.78 (0.69, 0.89) | <0.01 | 3.84 (3.52, 4.16) | 2.29 (1.72, 3.03) | <0.01 |
| Mother non-UK-born & SP non-UK-born | | |  |  |  |  |  |  |  |
| 1 Least deprived | 36.9 (35.7, 38.1) | Ref. |  | 74.4 (70.7, 78.1) | Ref. |  | 1.26 (1.05, 1.47) | Ref. |  |
| 2 | 35.8 (34.8, 36.8) | 0.97 (0.93, 1.01) | 0.19 | 70.8 (67.7, 73.9) | 0.95 (0.89, 1.01) | 0.13 | 1.84 (1.62, 2.07) | 1.46 (1.20, 1.78) | <0.01 |
| 3 | 37.5 (36.7, 38.4) | 1.02 (0.98, 1.06) | 0.37 | 67.2 (64.8, 69.6) | 0.90 (0.85, 0.96) | <0.01 | 2.49 (2.28, 2.71) | 1.98 (1.66, 2.36) | <0.01 |
| 4 | 40.0 (39.3, 40.7) | 1.09 (1.05, 1.12) | <0.01 | 63.2 (61.4, 65.1) | 0.85 (0.80, 0.90) | <0.01 | 3.04 (2.83, 3.25) | 2.41 (2.04, 2.85) | <0.01 |
| 5 Most deprived | 51.2 (50.5, 51.9) | 1.39 (1.34, 1.44) | <0.01 | 64.9 (63.2, 66.6) | 0.87 (0.83, 0.92) | <0.01 | 3.45 (3.24, 3.66) | 2.73 (2.32, 3.22) | <0.01 |
| Mother non-UK-born & SP UK-born | |  |  |  |  |  |  |  |  |
| 1 Least deprived | 41.3 (39.9, 42.6) | Ref. |  | 62.5 (59.0, 66.0) | Ref. |  | 0.73 (0.57, 0.89) | Ref. |  |
| 2 | 43.1 (41.6, 44.5) | 1.04 (1.00, 1.09) | 0.07 | 65.9 (62.2, 69.6) | 1.05 (0.98, 1.14) | 0.18 | 0.96 (0.77, 1.15) | 1.31 (0.98, 1.76) | 0.07 |
| 3 | 44.4 (43.0, 45.9) | 1.08 (1.03, 1.13) | <0.01 | 64.4 (60.9, 67.9) | 1.03 (0.95, 1.11) | 0.45 | 1.24 (1.04, 1.45) | 1.70 (1.30, 2.23) | <0.01 |
| 4 | 48.2 (46.8, 49.6) | 1.17 (1.12, 1.22) | <0.01 | 63.7 (60.5, 66.9) | 1.02 (0.95, 1.10) | 0.62 | 1.93 (1.69, 2.18) | 2.65 (2.06, 3.40) | <0.01 |
| 5 Most deprived | 65.4 (63.7, 67.1) | 1.58 (1.52, 1.65) | <0.01 | 60.3 (57.3, 63.3) | 0.96 (0.90, 1.04) | 0.33 | 2.98 (2.68, 3.29) | 4.08 (3.22, 5.18) | <0.01 |
| Mother non-UK-born (sole registration) | |  |  |  |  |  |  |  |  |
| 1 Least deprived | 40.5 (33.0, 48.1) | Ref. |  | 46.1 (26.9, 65.3) | Ref. |  | 0.33 (-0.32, 0.98) | Ref. |  |
| 2 | 44.6 (38.9, 50.4) | 1.10 (0.88, 1.38) | 0.40 | 48.3 (35.3, 61.4) | 1.05 (0.64, 1.72) | 0.85 | 3.43 (1.93, 4.92) | 10.31 (1.39, 76.62) | 0.02 |
| 3 | 44.9 (40.9, 48.9) | 1.11 (0.90, 1.36) | 0.33 | 54.1 (44.9, 63.4) | 1.17 (0.75, 1.84) | 0.48 | 2.90 (2.00, 3.80) | 8.73 (1.20, 63.36) | 0.03 |
| 4 | 44.0 (41.4, 46.6) | 1.09 (0.89, 1.32) | 0.40 | 42.1 (36.8, 47.4) | 0.91 (0.59, 1.41) | 0.68 | 3.86 (3.13, 4.60) | 11.63 (1.63, 83.10) | 0.01 |
| 5 Most deprived | 52.5 (50.1, 54.8) | 1.30 (1.07, 1.57) | 0.01 | 42.6 (38.4, 46.9) | 0.92 (0.60, 1.42) | 0.72 | 3.92 (3.35, 4.48) | 11.79 (1.66, 83.92) | 0.01 |

CI = Confidence interval, IMD = index of multiple deprivation, IRR = incidence rate ratio, SP=second parent; * results derived from negative binomial/Poisson regression models adjusted for year of birth, parental migration status, IMD group and parental migration status*IMD group interaction term (regression model results available on request); marginal incidence rates derived from models with year of birth set to mid-study (2011); IRR of admission rates for IMD groups in comparison to the least deprived IMD group, within parental migration status group; **negative binomial regression, *N* = 4,174,596, AIC = 4206508.56 (compared with AIC = 4206994.09 for model without interaction term); ***Negative binomial regression, *N* = 4,174,596, AIC = 4206508.56 with interaction term, AIC = 4206994.09without interaction term; ^a^Poisson regression, *N* = 2,973,284, AIC = 220,264 with interaction term, AIC = 220,433 without interaction term
