# Supplementary material for: Parental migration, socioeconomic deprivation and hospital admissions in preschool children in England: national birth cohort study, 2008 to 2014
Source: BMC Med. 2024 Sep 27;22:416. doi: 10.1186/s12916-024-03619-1 (PMC11438240; doi:10.1186/s12916-024-03619-1)
Supplement: Supplementary file 8 — Additional file 8. Sensitivity analyses results (Figures S2-S4). Fig S2—Estimated rates of emergency admissions stratified by London/non-London residence at birth. Fig S3—Estimated rates of emergency admissions stratified by emigration sensitivity analysis scenario. Fig S4—Estimated rates of planned admissions stratified by emigration sensitivity analysis scenario. [file 12916_2024_3619_MOESM8_ESM.docx]

# Additional File 8:
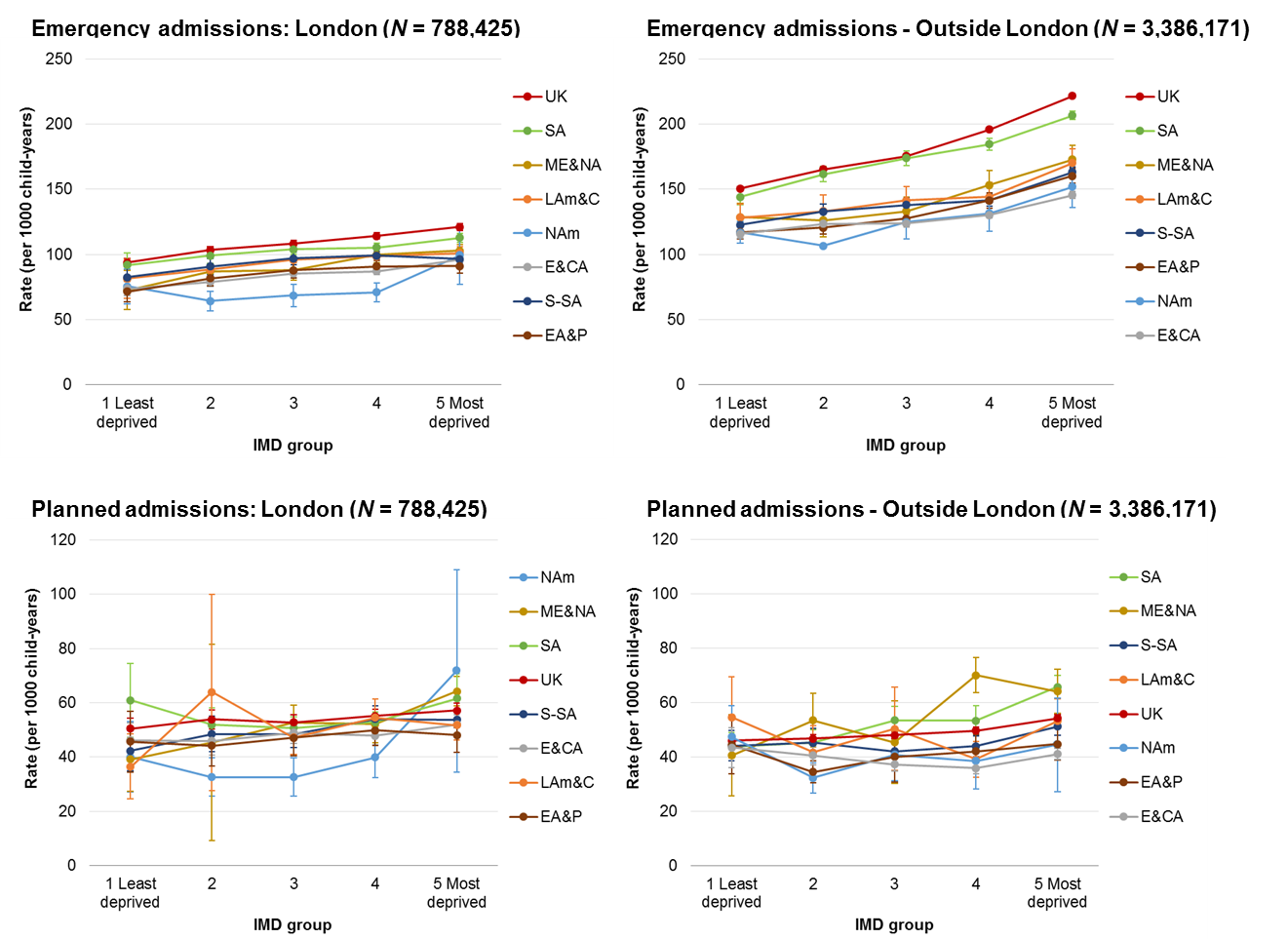
Results - sensitivity analyses

Figure S2. Estimated incidence rates* of emergency and planned hospital admissions (per 1000 child-years), by maternal region of birth and IMD group: stratified by London/non-London residence at birth. N = number of children included in each analyses; IMD= index of multiple deprivation; EA&P= East Asia and Pacific, E&CA= Europe (excl. UK) and Central Asia, LAm&C= Latin America and Caribbean, ME&NA= Middle East and North Africa, NAm= North America, SA= South Asia, S-SA= Sub-Saharan Africa; *with year of birth set to mid-study (2011) derived from negative binomial regression models adjusted for year of birth, maternal world region of birth, IMD group, and maternal world region of birth*IMD group interaction term.


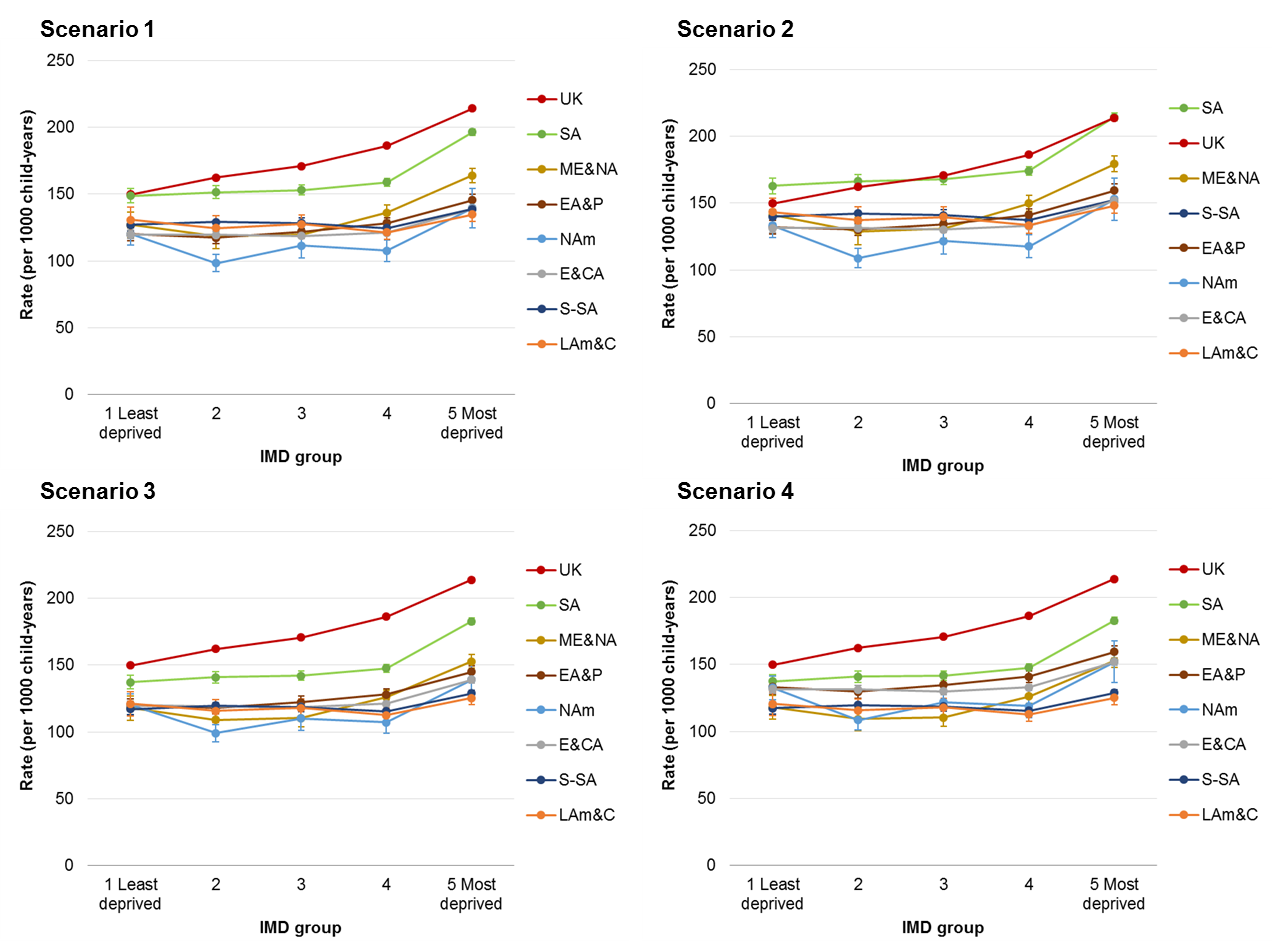
Figure S3. Estimated incidence rates* of emergency admissions to hospital (per 1000 child-years), by maternal region of birth: stratified by emigration sensitivity analysis scenario (see Additional File 3). IMD= index of multiple deprivation; EA&P= East Asia and Pacific, E&CA= Europe (excl. UK) and Central Asia, LAm&C= Latin America and Caribbean, ME&NA= Middle East and North Africa, NAm= North America, SA= South Asia, S-SA= Sub-Saharan Africa; *with year of birth set to mid-study (2011) derived from negative binomial regression models (*N* = 4,174,596) adjusted for year of birth, maternal world region of birth, IMD group, and maternal world region of birth*IMD group interaction term.


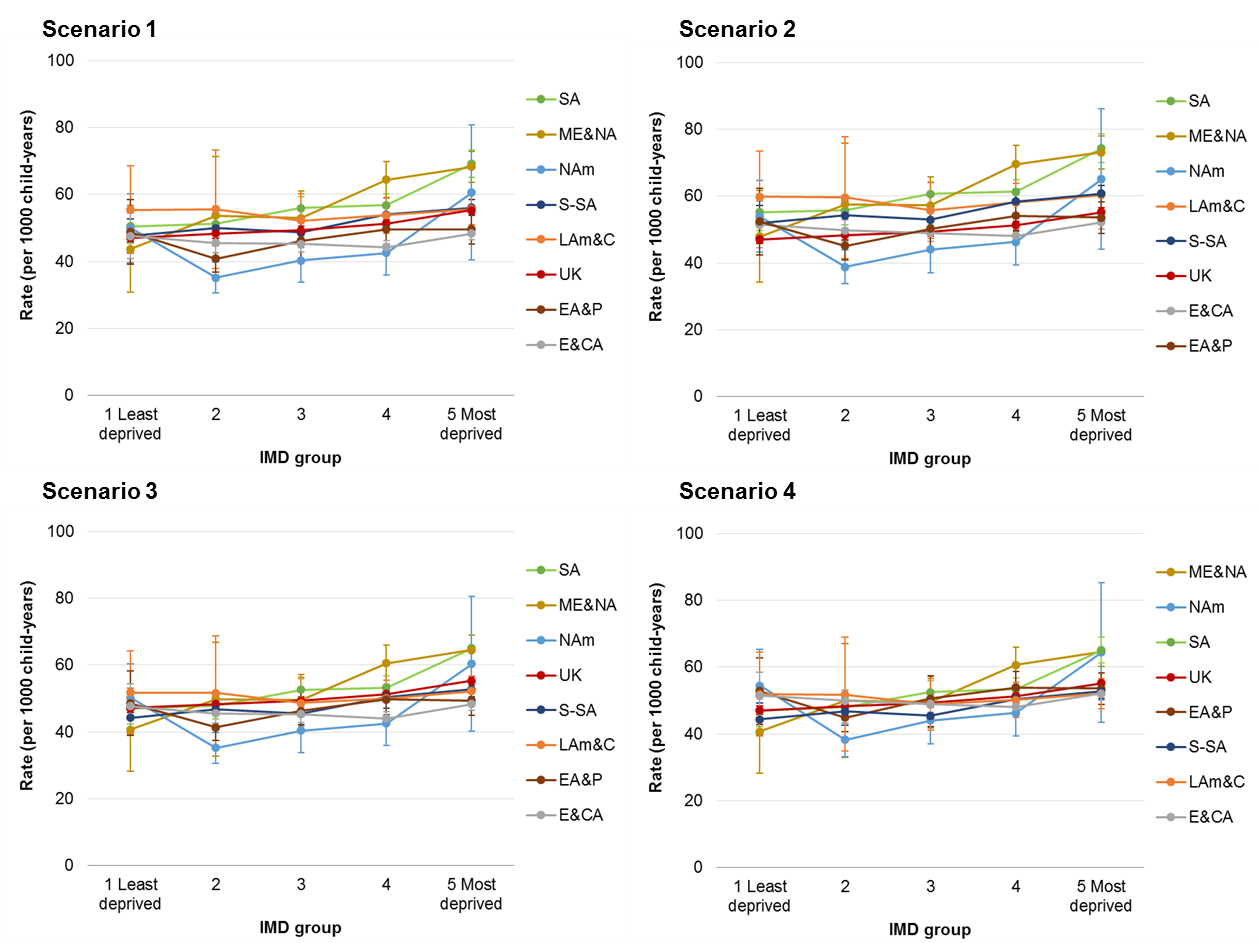


Figure S4. Estimated incidence rates* of planned admissions to hospital (per 1000 child-years), by maternal region of birth: stratified by emigration sensitivity analysis scenario (see Appendix C). IMD= index of multiple deprivation; EA&P= East Asia and Pacific, E&CA= Europe (excl. UK) and Central Asia, LAm&C= Latin America and Caribbean, ME&NA= Middle East and North Africa, NAm= North America, SA= South Asia, S-SA= Sub-Saharan Africa; *with year of birth set to mid-study (2011) derived from negative binomial regression models (*N* = 4,174,596) adjusted for year of birth, maternal world region of birth, IMD group, and maternal world region of birth*IMD group interaction term.
